# Novel function of TREK-1 in regulating adipocyte differentiation and lipid accumulation

**DOI:** 10.1038/s41419-025-07478-3

**Published:** 2025-03-08

**Authors:** Ajung Kim, Seoyeong Jung, Yongeun Kim, Jonghoon Jung, Soomin Lee, Hojin Lee, Min Jung Kim, Jae-Yong Park, Eun Mi Hwang, Jaekwang Lee

**Affiliations:** 1 Food Functionality Research Division, Korea Food Research Institute, Wanju, 55365 South Korea; 2 Brain Science Institute, Korea Institute of Science and Technology, Seoul, 02792 South Korea; 3 School of Biosystems and Biomedical Sciences, College of Health Sciences, Korea University, Seoul, 02841 South Korea

**Keywords:** Potassium channels, Obesity

## Abstract

K2P (two-pore domain potassium) channels, a diversified class of K^+^-selective ion channels, have been found to affect a wide range of physiological processes in the body. Despite their established significance in regulating proliferation and differentiation in multiple cell types, K2P channels’ specific role in adipogenic differentiation (adipogenesis) remains poorly understood. In this study, we investigated the engagement of K2P channels, specifically KCNK2 (also known as TREK-1), in adipogenesis using primary cultured adipocytes and TREK-1 knockout (KO) mice. Our findings showed that TREK-1 expression in adipocytes decreases substantially during adipogenesis. This typically causes an increased Ca^2+^ influx and alters the electrical potential of the cell membrane in 3T3-L1 cell lines. Furthermore, we observed an increase in differentiation and lipid accumulation in both 3T3-L1 cell lines and primary cultured adipocytes when the TREK-1 activity was blocked with Spadin, the specific inhibitors, and TREK-1 shRNA. Finally, our findings revealed that mice lacking TREK-1 gained more fat mass and had worse glucose tolerance when fed a high-fat diet (HFD) compared to the wild-type controls. The findings demonstrate that increase of the membrane potential at adipocytes through the downregulation of TREK-1 can influence the progression of adipogenesis.

## Introduction

Adipogenesis is a complex and highly regulated process during which preadipocytes differentiate into mature adipocytes. These cells not only store lipid droplets as triglycerides for future energy release but also participate in pathological processes, including obesity, insulin resistance, and metabolic disorders. In adipogenesis, it is primarily driven by two cellular mechanisms: hyperplasia and hypertrophy. These processes contribute to the development of obesity and related metabolic disorders. While hyperplasia refers to the increase in the number of adipocytes, hypertrophy is the increase in the size of existing adipocytes without an increase in their number. To initiate hyperplasia, multipotent mesenchymal stem cells (MSCs) are committed to the adipogenic lineage under appropriate stimuli (such as hormones or growth factors). This process so called commitment stage and is regulated by transcriptional factors including CCAAT/enhancer-binding protein(C/EBP) β and δ. Following the commitment stage, the cells undergo clonal expansion—a process predominantly observed in mouse-derived adipocyte cell lines—enabling proliferation prior to progressing to terminal differentiation. In this stage, Peroxisome proliferator-activated receptor (PPAR)γ, along with C/EBPα, drives the expression of adipocyte-specific genes, leading to lipid accumulation, changes in morphology, and acquisition of adipocyte functions such as lipid storage (fatty acid-binding protein 4 (FABP4, aP2)) and secretion of adipokines (e.g., leptin, adiponectin) [1–3]. Moreover, hormones, growth factors, and transcriptional regulators all play roles in adipogenesis, controlling gene expression and cell fate decisions [4, 5]. Membrane potentials are crucial for regulating cell proliferation, differentiation, and signaling, with potassium ion channels being an important component of the cellular machinery that governs membrane potential [6]. Specifically, K2P channels contribute to leaking K^+^ currents and help stabilize negative resting membrane potentials in many cell types [7]. Although some members of the K2P potassium channel family have been reported to be expressed in adipose tissue or adipocytes [8, 9], the expression changes, and mechanisms of K2P channels during adipogenesis remain poorly understood.

Adipocytes, the primary cells in adipose tissue, are essential in maintaining energy balance by storing excess energy or regulating thermogenesis. The number and size of these cells might fluctuate depending on a person’s diet and physical condition [10]. The differentiation of preadipocytes, into mature adipocytes is central to adipose tissue development and metabolism regulation [11]. Notably, stem cell proliferation is linked to obesity, which is defined by an increase in white adipose tissue (WAT) [12]. Ion channels have emerged as key components affecting the development of adipocytes. The determination of adipocyte fate is partially regulated by voltage-dependent potassium channels (e.g., TRPV, TRPM) and calcium channels (e.g., Cav3.1) [13]. These channels respond to changes in membrane potential by influencing calcium, an intracellular messenger crucial for adipocyte differentiation and proliferation. Compared to resting cells, proliferating or differentiating cells exhibit distinct ion channel properties and expression patterns, providing a new perspective on adipocyte differentiation and the onset of obesity. Voltage-gated K^+^ currents (Kv) have been identified in human adipose tissue and human adipose tissue-derived stem cells, suggesting their potential role in adipogenesis [14, 15]. Additionally, calcium signaling via voltage-gated calcium channels (VGCCs) in adipocytes is essential for adipogenesis, governing key functions such as proliferation, differentiation, and energy metabolism. Dysregulation of cytosolic calcium has been linked to metabolic abnormalities associated with human obesity [16].

In this study, we present results from experiments using cell lines, cultured primary adipocytes, and animal models induced by a high-fat diet. We also demonstrated experimentally that changes in membrane potential mediated by TREK-1 can induce alterations in intracellular calcium signaling via VGCC. Furthermore, transcriptome data confirmed that these changes were replicated during the differentiation of adipose stem cells derived from human adipose tissue [17]. The observed inhibition of adipocyte differentiation by TREK-1 indicates its potential as a promising therapeutic target for obesity. This offers a novel approach to modulating adipogenesis and may potentially reduce the development of adipose tissue.

## Materials and methods

### Chemicals

Spadin (Tocris, cat #5594) was purchased from TOCRIS (Tocris Bioscience, Bristol, UK) and stored as a stock solution at −20 °C, dissolved in DMSO. It was diluted to the required concentration in a standard bath solution immediately before experimentation. Nifedipine (Tocris, cat #1075) was stored as stock solutions at −4 °C and similarly diluted to the required concentrations in a standard bath solution immediately before use. ML402 (Tocris, cat #6888) was stored at −4 °C. Before the start of the experiment, it was diluted to the desired concentration and used. GO6983 (Tocris, cat #2285) was purchased from TOCRIS, stored as stock solutions at −20 °C, and diluted to the required concentrations in a standard bath solution immediately before experimentation.

### Cell culture and adipocyte differentiation

3T3-L1 preadipocytes were purchased from American Type Culture Collection (ATCC, #CL173) and subcultured in Dulbecco’s Modified Eagle Medium (Gibco, #11965092) with 10% (v/v) bovine serum (Gibco, #16170078) and 1% (v/v) penicillin/streptomycin (Gibco, #10378016). Cultures were maintained in a humidified 5% CO_2_ incubator at 37 °C. After reaching 80% confluence, 3T3-L1 preadipocytes were induced differentiation by replacing the medium with DMEM containing 10% FBS (Gibco, #16000044) plus 1 µg/ml insulin, 0.5 mM isobutyl-1-methylxanthine (IBMX) (Sigma-Aldrich, #I5879), 1 µM dexamethasone (Sigma-Aldrich, #D4902), and 2 µM rosiglitazone (Sigma-Aldrich, #R2408) for 3 days. Afterward, the medium was changed with DMEM containing 10% FBS plus 1ug/ml insulin (Sigma-Aldrich, #I6634), and the cells were further cultured for 2 or 4 days.

### Primary adipocyte culture

Primary white adipocytes were isolated from the inguinal WAT of 6–8-week-old C57BL/6 mice. The extracted WAT was washed in a 15 mL tube. An enzyme solution was prepared by mixing Type II Collagenase (Worthington Industries, #SL004176), Dispase II (Roche Diagnostics, #11760200), and PBS (Gibco, #14190250). A 0.5 g sample of tissue was incubated in 1.5 mL of enzyme solution at 37 °C in an incubator for 1 h. After filtering, the solution was transferred to a new 15 mL tube and centrifuged at 2200 rpm for 10 min. The supernatant was removed, and red blood cells were lysed using ABC Lysis Buffer (Gibco, #A1049201), followed by a second centrifugation at 2200 rpm for 10 min. The supernatant was again discarded, and the adipocytes were resuspended in DMEM/F12 (Welgene, #LM002) with 10% (v/v) NBCS (Gibco, #26010074). After the final centrifugation and removal of the supernatant, the adipocytes were transferred to a cell culture flask containing the DMEM/F12 mixture. The flask was incubated at 37 °C in a CO_2_ incubator, and the medium was changed after approximately 3 days.

### Real-time PCR

Total RNA was isolated from ND and D5 cells using an RNA purification kit (GeneAll) according to the manufacturer’s instructions. The cDNAs were synthesized from 1 µg total RNA through reverse transcription using a SensiFAST^TM^ cDNA Synthesis Kit (BIOLINE) according to the manufacturer’s instructions. Real-time PCR was performed using a SensiFAST^TM^ Probe Hi-ROX kit (BIOLINE, London, UK). Primer sets for KCNK1, KCNK2, KCNK3, KCNK4, KCNK5, KCNK6, KCNK9, KCNK10, KCNK12, KCNK13, KCNK16, KCNK18 (Supplementary Table 1), and GAPDH were purchased from IDT (PrimeTime qPCR assays). GAPDH was used as the reference gene. The 2^−ΔΔCt^ method was used to calculate fold changes in gene expression. All experiments were repeated at least three times.

### Western blot

Cells were treated with the samples, collected, washed twice with cold PBS, and lysed in cell lysis buffer (Cell signaling Biotechnology, cat #9803S) on ice for 10–15 min. The lysate was clarified by centrifugation, and protein concentration was measured using a Pierce™ BCA protein assay kit (Thermo Scientific, cat #23225) following the manufacturer’s instructions. For western blotting, cells were lysed with lysis buffer (1% NP-40, 0.5% Sodium deoxycholate, 0.2% SDS, 150 mM NaCl, and 50 mM Tris) containing a protease inhibitor cocktail, pH 7.5). Total proteins were subjected to SDS-PAGE and transferred to PVDF membranes. The membranes were blocked with 5% non-fat milk and then blotted with the appropriate antibodies: anti-TREK-1 (Santa Cruz, cat #sc-11556), anti-PPARɣ (Cell Signaling Technology, cat #2435), anti-C/EBPɑ (Cell Signaling Technology, cat #2295), anti-p-ERK (Cell Signaling Technology, cat #4370), anti-ERK (Cell Signaling Technology, cat #4695), anti-p-PKCɑ (Santa Cruz, cat #sc-377565), anti-PKCɑ (Abcam, cat #ab4124), anti-p-AMPK${\rm{\alpha }}$ (Cell Signaling Technology, cat #2535S), anti-AMPK${\rm{\alpha }}$ (Cell signaling Technology, cat #2793S), anti-FABP4 (Cell Signaling Technology, cat #2120), anti-Actin (Sigma-Aldrich, cat #A5441). The membranes were then washed and incubated with HRP-conjugated secondary antibodies (BETHYL, cat #A120-101P/ #A90-116P). The blots were detected using an ECL Western Blotting Substrate (Thermo Fisher Scientific, cat #34580). Western Blot Band intensity was quantified using ImageJ software (Fiji, ImageJ).

### Lipid staining and analysis (Oil red O staining)

Lipid staining was performed using Oil Red O (ORO) (Sigma-Aldrich, cat #O0625). ORO dye was dissolved in isopropanol and free water to stain the loaded lipid droplets on the adipocytes, which were then fixed with 4% formaldehyde for 15 min. Next, the cells were washed twice with PBS, and adipocytes were incubated with ORO solution at room temperature for 15–30 min. The cells were then washed twice with 0.1 M PBS for 5 min. Bright field images are taken by microscope digital camera (Olympus, cat #DP73). ORO dye was dissolved by isopropanol to quantify the relative Tryglyceride accumulation by measuring absorbance at 510 nm using microplate reader (Biotek, Epoch).

### Electrophysiology

Whole-cell voltage-clamp recordings were performed using a MultiClamp 700 B amplifier (Molecular Devices, MultiClamp700B) using the pClamp10 software (Molecular Devices, pClamp/AxoScope 10). Whole-cell patch-clamp recordings were performed using glass pipettes prepared with a 2-stage vertical pipette puller (P-1000, Shutter Instruments). The pipette resistance value was between 5 and 10 MΩ. For recording potassium current, the pipettes were filled with a solution having the following composition (in mM): 140.0 K-gluconate, 10.0 HEPES, 0.5 EGTA, 10.0 glucose, 2.0 Na-ATP, and 0.5 Na-GTP; pH was adjusted to 7.2. standard solution of the pipetted sample (mM). The current-voltage (I–V) relations were measured by applying 1 s ramp pulses (from −60 mV to +140 mV) from a holding potential at −40 mV. The Digidata 1440 A interface (Molecular Devices, Digidata 1440 A) was used to convert digital to analog signals between the amplifier and the computer. Whole-cell currents were recorded 15–20 min after changing the Spadin-containing solution.

### Calcium imaging

3T3-L1 preadipocyte were seeded on 96well plate at 2 × 10^4^ cells/well density. Intracellular Ca^2+^ concentration was monitored by loading cultured 3T3-L1 preadipocytes with fluo-4AM dye (Thermo Fisher, Carlsbad, cat #14201). 3T3-L1 was incubated with 5 μM Fluo-4AM in medium and incubated for 30 min. was used in the experiments within 1 h. The bath solution contained 140 mM NaCl, 5 mM KCl, 2 mM MgCl_2_, 2 mM CaCl_2_, 10 mM HEPES, and 10 mM glucose at pH 7.4. Imaging was performed using a microscope to detect changes in fluorescence signals.

### Generation and genotyping of TREK-1 knock-out mice

TREK-1 knock-out (KO) mice were generated using the CRISPR/Cas9 system. Briefly, Cas9 mRNA and sgRNA were microinjected into the fertilized embryos of C57BL/6 mice (Korea Bio Co., Inc., Korea). Mutations in Kcnk2 were confirmed using sequencing (Korea Bio Co., Inc.). TREK-1 KO mice were genotyped by genomic DNA PCR analysis using genomic DNA prepared from their tails. Wild-type littermates were used as controls. TREK-1 KO forward: 5’-aatccttagccatccatgc-3,’ TREK-1 KO reverse: 5’-ctatctcagatggctttgacc-3.’ (Supplementary Fig. 1).

### Animals and high fat diets

Seven-week old male C57BL/6J WT and TREK-1 KO mice were used in this study. Animal care and handling were performed according to the institutional guidelines of Institutional Animal Care and Use Committee (IACUC, KIST-2020-068) at the Korea Institute of Science and Technology (Seoul, Korea). The mice were housed in a temperature (22–26 °C) and humidity-controlled animal room and maintained on a 12-h light/ 12-h dark cycle (light from 07:00 a.m. to 07:00 p.m.) with food and water provided during the experiments. One week before the high-fat diet, the mice were randomly distributed into the following four groups: (i) control mice fed a normal chow diet (10 kcal% fat content; Research Diets, D12450B); (ii) mice fed an HFD (60 kcal% fat content; Research Diets, D12492); (iii) TREK-1 KO mice fed a normal chow diet; (iv) TREK-1 KO mice fed an HFD. This study was conducted over 13 weeks. Food consumption and body weight were measured twice a week. Total calorie intake was calculated by the sum of calories consumed from food. NMR (Minispec LF90II, Bruker Bioscience Corp.) was used to investigate body composition.

### Oral glucose tolerance test (OGGT)

Two days before euthanasia, mice were fasted for 12 h prior to the test to standardize baseline glucose levels. Following fasting, each mouse was administered a glucose solution (2 g/kg body weight) by oral gavage. Blood glucose levels were measured at baseline (0 min) and at intervals of 15, 30, 60, 90, and 120 min post-gavage. Blood samples were collected from the tail vein, and glucose levels were measured using a glucometer (Accu-Chek, Roche). The glucose tolerance was evaluated by analyzing the glucose levels over time and calculating the area under the curve (AUC) for each mouse.

### Plasma leptin (ELISA)

Plasma leptin levels were quantified using a mouse leptin quantitative ELISA kit (R&D Systems, MOB00B), which was used according to manufacturer’s instructions. Plasma samples were prepared and diluted as recommended in the kit instructions. Each sample and standard were added to the designated wells of a 96-well microplate pre-coated with a capture antibody specific to mouse leptin. The plate was then incubated according to the protocol to allow leptin binding. After washing to remove unbound substances, a detection antibody was added, followed by another incubation and washing step. A substrate solution was added to each well, developing a color reaction proportional to the leptin concentration in the sample. The reaction was stopped, and absorbance was measured at the specified wavelength using a microplate reader. Leptin concentrations were calculated based on the standard curve generated with known leptin concentrations.

### H&E staining and analysis

iWAT samples were collected for histological analyses. Immediately after harvesting, the tissue was washed with saline, placed in histological cassettes, and fixed in 10% neutral buffered formalin solution. After 24 h, tissues were processed and embedded in paraffin. Multiple Section (4 µm) were prepared and stained with hematoxylin and eosin (H&E) for morphological analysis. Images were captured using a Leica microscope (Leica DMi8). AdipoCount (csbio, version 1.0) was used to measure the cell count and size of adipocytes [18].

### shRNA transfection

A shRNA against TREK-1 was used as previously reported [19]. Briefly, TREK-1 shRNA was transfected into iWAT SVC 24 hours before induction of adipogenic differentiation. Transfection was carried out using the Transfection Reagent Lipofectamine 2000 (Invitrogen, cat #11668019). For a 6-well plate, 5 ul of Lipofectamine 2000 was used to transfect cells with 1ug total shRNA according to the manufacturer’s instructions. To enhance transfection efficiency, CombiMag Transfection Reagent (OZBIOSCIENCES, cat #CM20200) was also used. Following adipogenic differentiation induction, electrophysiology and ORO staining were performed to analyze RMP (resting membrane potential), lipid accumulation, and Western blot analysis was conducted.

### Statistical analysis

The data are represented by the mean ± SEM. The importance of comparing two groups was determined using Student’s *t* test, and the importance of multiple comparisons (more than two groups) was evaluated by one-way ANOVA with a post-hoc test. Two-way analysis of variance (ANOVA) and Tukey’s post-hoc tests were used for experiments with two independent variables. The significance levels are specified as *P < 0.05, **P < 0.01, ***P < 0.001 and ****P < 0.0001.

## Results

### Reduction in K^+^ currents and increase in membrane potentials observed in adipogenesis in 3T3-L1 cell line

**Fig. 1:**
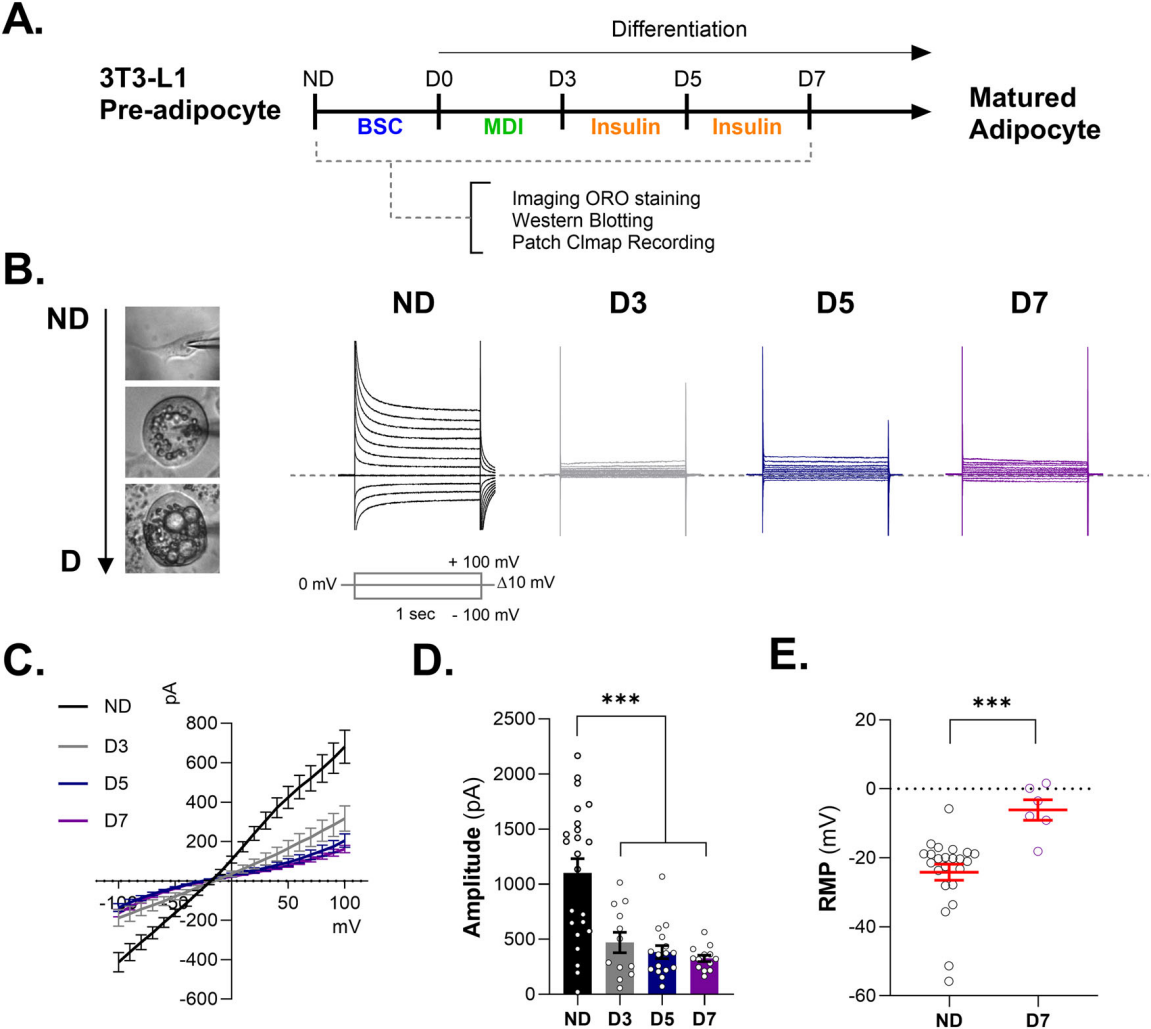
K^+^ currents and membrane potentials in 3T3-L1 cell line during adipogenesis. **A** Schematic experimental design. 3T3-L1 preadipocyte were differentiated into mature adipocyte for 7 days. Imaging ORO staining, western blotting, and patch clamp recording were conducted. **B** representative images of 3T3-L1 cell morphology (left). The cells were used for measuring K^+^ channel conductance using voltage-clamp recording at ND, D3, D5, and D7 (right). Command voltage pulse protocol: voltages were stepped from −100 mV to +100 mV in increments of 10 mV from a holding potential of −40 mV. The dotted line indicates 0 pA. **C** The current–voltage relationship (I–V) from −100 mV to +100 mV. Representative whole-cell passive conductance of adipocytes in preadipocytes and mature adipocytes through the 3T3-L1 cell line. **D** Bar graph showing the amplitude of each condition from −100 mV to +100 mV through preadipocytes and mature adipocytes. Data are mean ± s.e.m. One-way ANOVA to obtain *p*-values. ***P < 0.001. The number of slices tested in at least 12 independent cells is indicated in each bar. **E** Resting membrane potential (RMP) on 3T3-L1 comparison before and after differentiation. Data are mean ± s.e.m. *P*-values were obtained with Student’s *t* test. ***P < 0.001.

We investigated the modulation of K^+^ channels during adipogenic differentiation (Fig. 1A). Using voltage-clamp recording techniques, we confirmed the expression of functional K^+^ channels in adipocytes and found that K^+^ current conductance gradually decreased after adipocyte differentiation at D3, D5, and D7 in 3T3-L1 adipocytes (Fig. 1B–D). The resting membrane potential (RMP) of 3T3-L1 adipocytes was significantly increased after adipocyte differentiation (Fig. 1E). These results showed that the membrane potential depolarized while preadipocyte differentiated into mature adipocytes.

### Adipogenic differentiation modulates the functional expression of TREK-1 in adipocytes

**Fig. 2:**
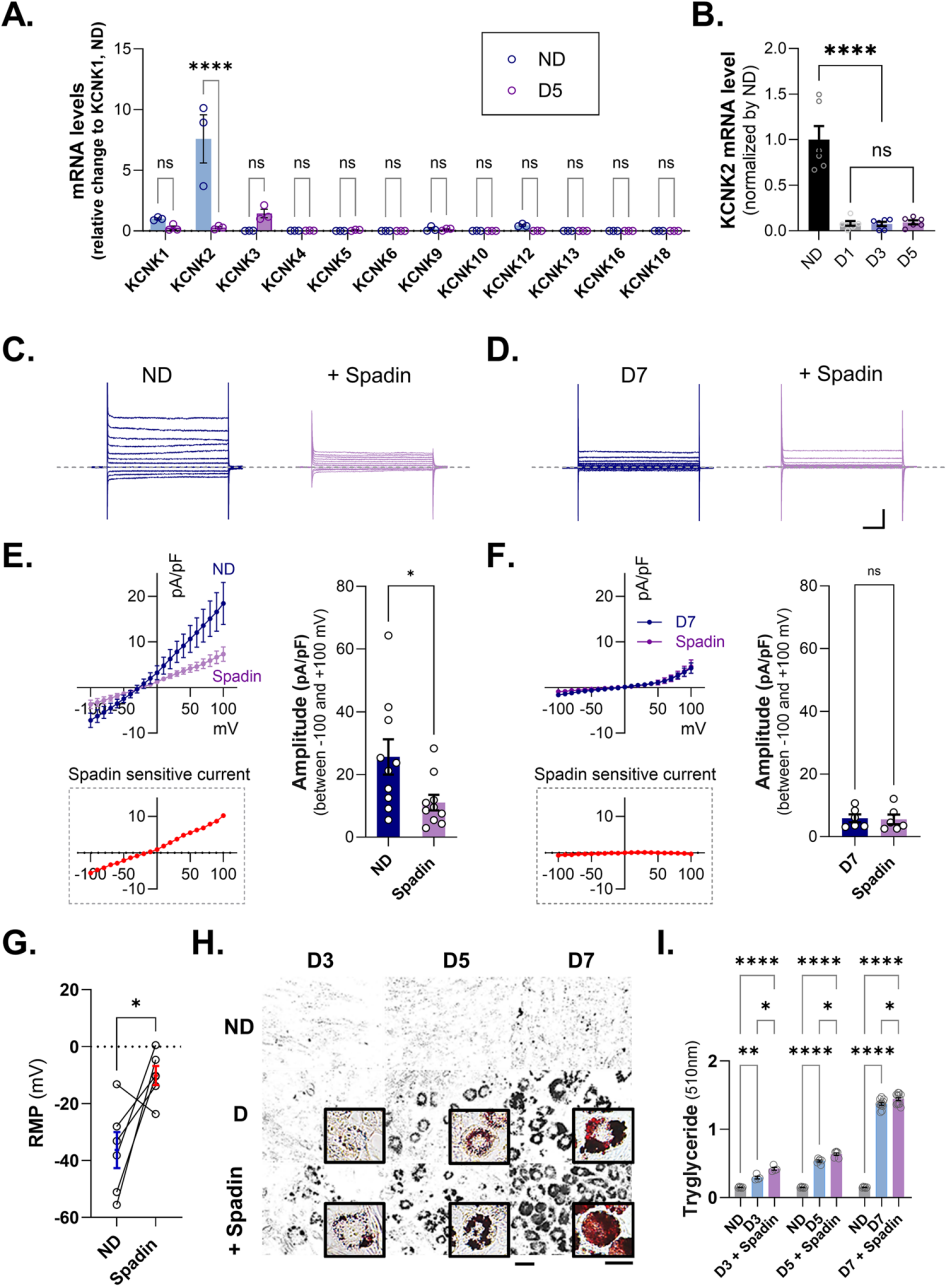
Potassium current and membrane potentials with TREK-1 antagonist treatment in 3T3-L1 cell line while adipocytes are differentiating. **A** The mRNA expression of KCNK families in ND and D5 during adipocyte differentiation. **B** Expression of KCNK2 mRNA by time of differentiation. **C** Representative traces of K+ current recordings for Spadin (1 μM) treatment in 3T3-L1 preadipocytes (left), Spadin treatment (right). **D** Representative traces of differentiated adipocyte K+ current recordings. Differentiation (left) and Spadin treatment (right). **E**, **F** Current densities induced by voltage ramping were recorded in differentiated adipocytes (blue) and Spadin-treated (purple). Spadin-sensitive current (red). **G** RMP on 3T3-L1 comparison before and after Spadin (1 μM) treatment. **H** Oil Red O staining image to measure cellular lipid droplets. Scale bars indicate 20 μm and 10 μm, respectively. **I** Quantification of ORO staining in 3T3-L1 cells treated with Spadin from D3 to D7. Data are mean ± s.e.m. *P*-values were obtained with one-way ANOVA ****P < 0.0001.

Next, we aimed to identify the ion channels that reduce the cell membrane potential during adipocyte maturation. Previous studies have shown that K2P channels are important for maintaining cell membrane potential, and changes in the expression of K2P channels have been confirmed using a transcriptome database (GSE227819) that compared the differentiation process of preadipocytes. The change in TREK-1 was the most significant among the K2P channels (Supplementary Fig. 2). Real-time PCR analysis revealed that the mRNA levels of KCNK2 (also known as TREK-1), a member of the K2P family, were predominantly expressed in 3T3-L1 preadipocytes cell line and were significantly reduced on day 5 after the induction of adipocyte differentiation (Fig. 2A). In more detail, the mRNA expression of K2P channels compared before and after differentiation showed that only TREK-1 was significantly changed, while KCNK5, 6, 10, and 16 were barely measured, and the other channels were also expressed to a very small extent. To further investigate the differentiation stage-specific expression of KCNK2, an examination of the mRNA expression of KCNK2 at ND, D1, D3, and D5 was conducted. It was observed that KCNK2 expression decreased significantly from D3, which represents the early stage of differentiation induction (Fig. 2B). These results suggest that K^+^-conductive ion channels, particularly TREK-1, may play a role in adipogenic differentiation by altering their properties or being downregulated during the process. Following a significant reduction in TREK-1 expression during adipocyte differentiation, we aimed to determine whether this reduction was due to downregulation of TREK-1 in 3T3-L1 preadipocyte cell line. To verify the functional expression of TREK-1, we measured the K^+^ currents in pre-differentiated (ND) and mature (D7) adipocytes. In the presence of 1 μM Spadin, a TREK-1 antagonist, voltage-step currents were significantly decreased in ND but not in D7 (Fig. 2C–F). Spadin-sensitive currents measured in both pre- and mature adipocytes suggested that the majority of the reduction in whole-cell currents were due to TREK-1 function. Moreover, we found that the inhibition of TREK-1 by Spadin induced the depolarization of RMP in preadipocytes (Fig. 2G), indicating that adipogenic differentiation modulates the functional expression of TREK-1 and RMP in adipocytes. To investigate the phenotypic effects of TREK-1 on adipogenesis, we evaluated cytosolic lipid accumulation using Oil Red O (ORO) staining. We observed that the inhibition of TREK-1 by Spadin treatment significantly increased lipid droplet formation during adipogenic differentiation (Fig. 2H, I). These findings suggested that the absence of TREK-1 makes adipocytes more susceptible to differentiation into mature cells.

### Adipogenic biomarkers were upregulated by TREK-1 inhibition in adipocytes

**Fig. 3:**
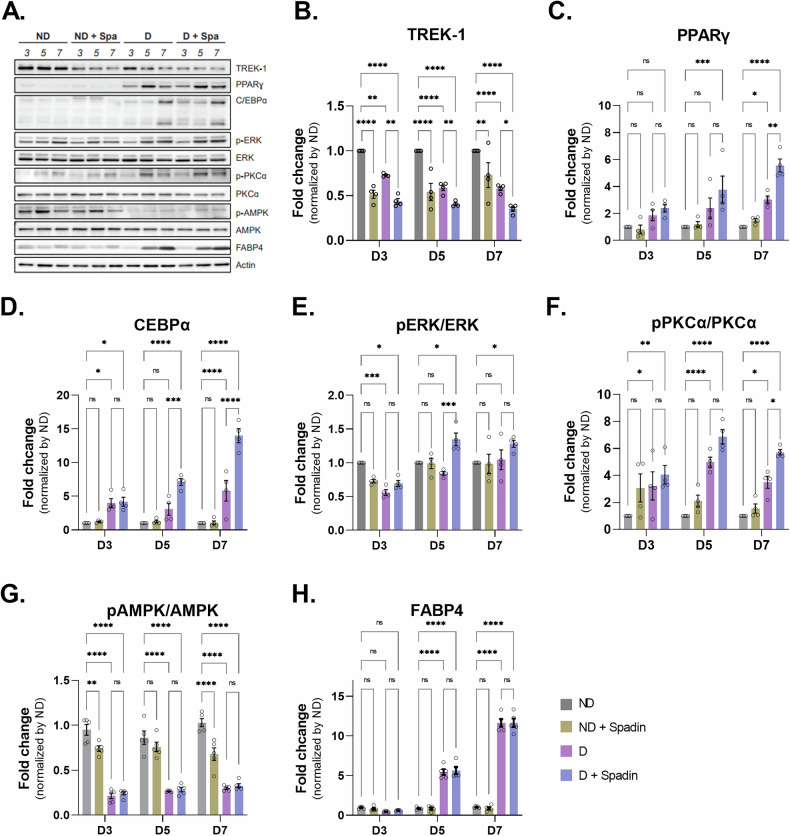
Alteration of adipogenic markers with TREK-1 antagonist in 3T3-L1 cell. **A** Adipogenic differentiation was induced in 3T3-L1 cell line. Protein expression were evaluated by WB analysis using antibodies specific for **B** TREK-1, **C** adipogenesis marker, PPARγ and **D** C/EBPα. **E** Expression enhancement induction protein protein (p-ERK, ERK). **F** Glucose transport protein (p-PKCα, PKCα). **G** Energy metabolism regulator, p-AMPK. **H** A marker for late adipogenic differentiation, FABP4.

Previous experiments have shown that a reduction in TREK-1 levels occurs during the induction of adipocyte differentiation. Furthermore, the inhibition of TREK-1 activity with Spadin during differentiation has been shown to enhance the process of adipogenesis. Consequently, we sought to ascertain whether this inhibition of TREK-1 activity induces acceleration of adipogenesis by examining alterations in the expression of various adipogenesis markers. To investigate the impact of TREK-1 inhibition on adipogenic differentiation, we performed western blot analysis to detect changes in adipogenic markers after differentiation induction with Spadin treatment (Fig. 3A). Initially, we confirmed the downregulation of TREK-1 following differentiation, and Spadin treatment further enhanced this downregulation (Fig. 3B). Our findings revealed a significant upregulation of PPARγ and C/EBPα expression upon inhibition of TREK-1 by Spadin treatment, indicating a potential regulatory role of TREK-1 in these adipogenic markers (Fig. 3C, D). In addition, we checked the expression of PPARγ2 among PPAR isoforms and found that a trend towards increased expression at D3 and D5, but by D7 its expression was significantly increased in the Spadin-treated group (Supplementary Fig. 3). Previous studies have indicated that the mitogen-activated protein kinase (MAPK) signaling pathway, especially the extracellular signal-regulated kinase (ERK) pathway, is a critical regulator of adipogenesis. Additionally, protein kinase C (PKC), an upstream modulator of ERK, is implicated in adipogenic processes and has been shown to influence both glucose transport and insulin resistance in adipocytes [20, 21]. It was observed that there was a tendency for the expression of phosphorylated PKCα to increase with Spadin treatment on day 5. However, on day 7, the PKCα phosphorylation was significantly increased by Spadin treatment. While the level of phosphorylated ERK exhibited a moderate elevation on day 5 in the presence of Spadin, the phosphorylated ERK expression did not demonstrate a notable increase in the presence of Spadin (Fig. 3E, F). To identify an alternative mechanistic pathway of TREK-1 in adipogenesis, we measured the AMPK, which is a well-known modulator of adipogenesis. During adipogenesis, a significant decrease in AMPK levels was observed, and this was not further reduced by Spadin treatment (Fig. 3G). Furthermore, we detected upregulation of FABP4, a late marker of adipogenic differentiation, during adipocyte differentiation, which was not further increased by Spadin treatment (Fig. 3H). In contrast, after silencing TREK-1 expression in preadipocyte 3T3-L1 cells using shRNA and inducing differentiation, the expression changes of adipogenic markers were observed at D5. The expression of PPARγ and C/EBPα increased significantly, similar to the treatment with Spadin, while a significant increase in the expression of FABP4 was observed that was not detected after Spadin treatment (Supplementary Fig. 4). Interestingly, we examined the relationship between TREK-1 and the expression of C/EBPβ, a marker involved in early differentiation, and found an increase in the expression of liver activating protein (LAP), one of the isoforms of C/EBPβ, upon Spadin treatment in the 3T3-L1 cell line (Supplementary Fig. 5). Overall, our results suggest that the inhibiting of the TREK-1 channel by Spadin treatment in 3T3-L1 adipocytes modulates the expression of key biomarkers involved in the initial stage of adipogenic differentiation.

### Inhibition of TREK-1 promotes adipogenesis via induction of intracellular Ca^2+^ increase through VDCC

**Fig. 4:**
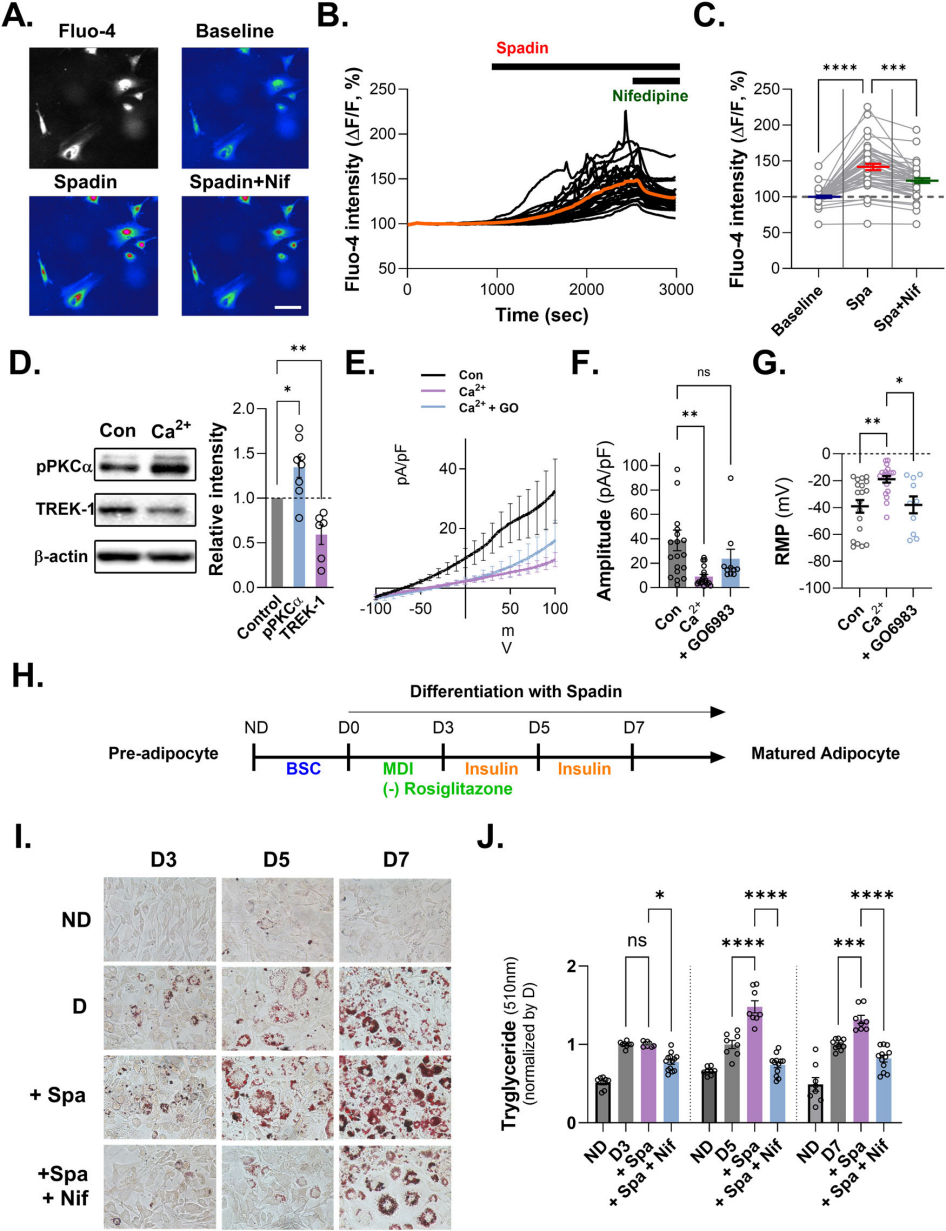
Calcium efflux with TREK-1 inhibitor or/and Ca^2+^ channel antagonist in 3T3-L1 cell line. **A** Calcium imaging of preadipocyte cells. **B** Calcium fluorescence of cultured 3T3-L1 during bath application of Spadin (1 μM) and nifedipine (10 μM). **C** Fluo-4 intensity in calcium imaging. Data are mean ± s.e.m. *P*-values were obtained with one-way ANOVA ****P < 0.0001. **D** Relative intensity of p-PKCα and TREK-1 upon calcium treatment in preadipocytes. **E** The current–voltage relationship (I–V) from −100 mV to +100 mV in preadipocytes treated with 5 mM Ca^2+^ and PKC inhibitor (GO6796). **F** Amplitude for potassium current (pA/pF). Data are mean ± s.e.m. *P*-values were obtained with one-way ANOVA **P < 0.01. **G** Changed resting membrane potential (RMP) upon calcium treatment and PKCα inhibitor treatment in preadipocytes. Data are mean ± s.e.m. *P*-values were obtained with one-way ANOVA ***P < 0.001. **H** Schematic outline of the experimental design. **I** ORO staining and morphological changes in 3T3-L1 cell, before and after Spadin treatment. **J** Quantification of ORO staining in 3T3-L1 cells treated with Spadin and nifedipine from D3 to D7. Data are mean ± s.e.m. *P*-values were obtained with one-way ANOVA ****P < 0.0001.

We showed adipogenic markers, including C/EBPα and PPARγ, which were increased upon Spadin treatment in differentiated adipocytes. Moreover, we found that the phosphorylated form of PKCα was elevated following Spadin treatment. Interestingly, we observed that the membrane potential of adipocytes was significantly depolarized during cell differentiation. Based on these findings, we hypothesized that the inhibiting of TREK-1 during adipogenic differentiation could lead to an increase in cytosolic Ca^2+^, an essential molecule in adipogenesis [22–29]. Calcium imaging experiments were performed to test this hypothesis using Fluo-4-loaded preadipocytes and an epifluorescence microscope. We observed that Ca^2+^ signals were evoked by the application of Spadin and significantly blocked by the co-application of nifedipine, an L-type calcium channel blocker (Fig. 4A–C). While this was not a complete inhibition of the Ca^2+^ increase, these results partially support our hypothesis that downregulation of TREK-1 during adipogenic differentiation can lead to an increase in cytosolic Ca^2+^ in adipocytes, which is critical for adipogenesis.

Given the reported dynamic regulation of TREK-1 activity by PKC, we sought to investigate whether PKC similarly modulates TREK-1 activity in adipocytes [30]. To further understand the effect of increased Ca^2+^ on PKC and TREK-1, we examined the expression levels of PKC and TREK-1 in the presence of high Ca^2+^ levels in the media. The activation of PKC, which has been reported to inhibit TREK-1 [30, 31], aligns with our finding that phosphorylated PKC was upregulated while TREK-1 was downregulated under high Ca²^+^ stimulation in adipocytes (Fig. 4D). We verified this protein change using whole-cell patch-clamp experiments, which showed that the K^+^ current in preadipocytes was reduced. The RMP was depolarized in the presence of high Ca^2+^ stimulation. The PKCα inhibitor, GO6983, blocked the reduction of the K^+^ current and RMP depolarization in high Ca^2+^-stimulated preadipocytes (Fig. 4E–G). To investigate the impact of increased Ca^2+^ on adipogenic differentiation, we examined whether the inhibition of Ca^2+^ increase, mediated by TREK-1 downregulation via VDCCs, could inhibit phenotypic changes, such as lipid accumulation during adipogenic differentiation, using ORO staining in 3T3-L1 cells. To isolate the effect of TREK-1 inhibition on adipogenesis, we cultured 3T3-L1 cells in an MDI medium without rosiglitazone to slow differentiation, as Spadin treatment accelerated adipogenic differentiation. We found that Spadin-induced lipid accumulation and adipogenic biomarkers increased at D5 and D7 compared to that in the control, and this increase was blocked by co-treatment with nifedipine (Fig. 4H–J and Supplementary Fig. 6). This Taken together, our findings demonstrate that the inhibition of TREK-1 promotes adipogenesis through an increase in intracellular calcium levels via VDCCs.

### TREK-1 activation prevented adipogenesis

**Fig. 5:**
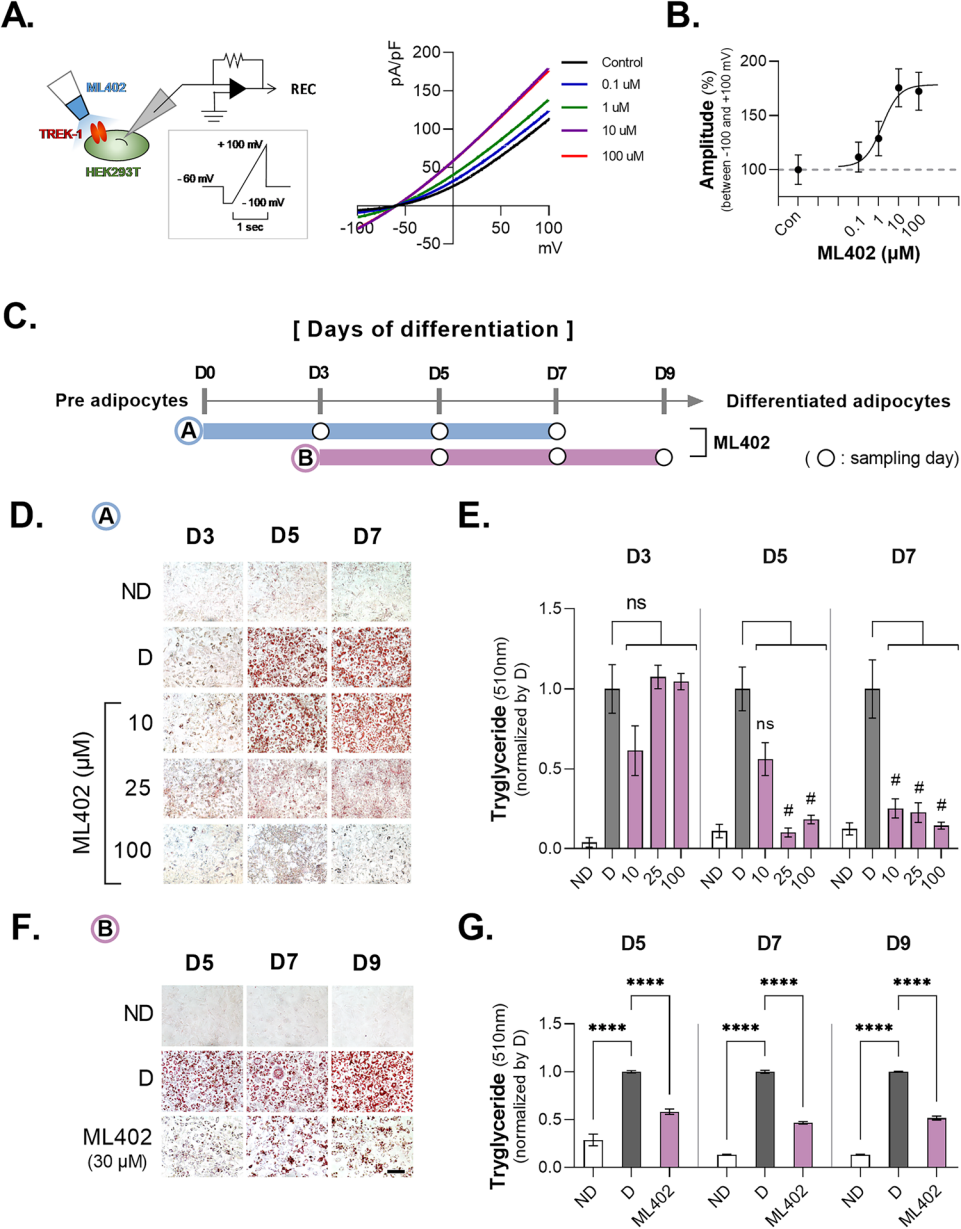
Adipogenesis in activation of TREK-1 in 3T3-L1 cell line. **A** Electrophysiology protocols. Schematic representation of the voltage ramp protocol used for HEK293T cells. colors indicate the cells transfected with a green fluorescent protein (GFP). step voltage protocol (holding potential −60 mV, 1 s steps ranging from −100 to +100 mV, spaced 10 mV. **B** Potassium current increases due to flow of ML402 set to 0.1 μM, 1 μM, 10 μM, 100 μM. **C** ML402 Treatment and Sampling Schedule. **D** Oil Red O imaging and morphological changes of 3T3-L1 cells upon long-term treatment of ML402. **E** OD values of 510 nm for Oil Red O during long-term treatment with ML402 (30 μM). One-way ANOVA to obtain *P*-values ****P < 0.0001. Data are mean ± s.e.m. **F** Oil Red O imaging and morphological changes of 3T3-L1 cells upon short-term treatment with ML402. **G** OD values at 510 nm for Oil Red O upon short-term treatment with ML402. One-way ANOVA to obtain *P*-values ****P < 0.0001. Data are mean ± s.e.m.

Based on our finding that the inhibition of TREK-1 promotes adipogenesis, we investigated the effect of TREK-1 activation on adipocyte differentiation using the TREK-1 and TREK-2 agonist, ML402. First, we determined the concentration of ML402 required to activate TREK-1 by whole-cell patch clamping of HEK293 cells fully transfected with TREK-1 (Fig. 5A). Although previous experiments have indicated that the expression of TREK-2 in the 3T3-L1 cell line is relatively low (Fig. 2A), the use of a heterologous system was employed as it is likely to have an effect. Our results revealed that over 10 µM ML402 was required to saturate the increase in TREK-1-mediated currents (Fig. 5B). We then divided the cells into two groups to evaluate the preventive and therapeutic effects of TREK-1 activation on adipocyte differentiation (Fig. 5C). In the first group, ML402 was administered at an early stage of differentiation induction. We observed a significant reduction in lipid accumulation at all doses on day 7 (D7), and ORO staining showed no difference compared to the untreated group on day 3 (D3) (Fig. 5D, E). In D3, mRNA expression of PPARγ has significant differences by ML402 treatment, but not in expression of C/EBPα using qPCR (Supplementary Fig. 7A). In the second group, ML402 was administered 3 days after the induction of differentiation. We observed a significant reduction in lipid accumulation on days 5, 7, and 9 (D5, D7, and D9) compared to that in the untreated group (Fig. 5F, G). Although mRNA expression of PPARγ and C/EBPα in day 5 (D5) has no remarkable decline by ML402, it showed ML402 decreased the mRNA expression of adipose markers (PPARγ and C/EBPα) in D7 and D9 (Supplementary Fig. 7B). These results suggested that activating TREK-1 in adipocytes has preventive and therapeutic effects on adipogenesis.

### TREK-1 knockdown promotes adipogenesis in iWAT Adipocytes

**Fig. 6:**
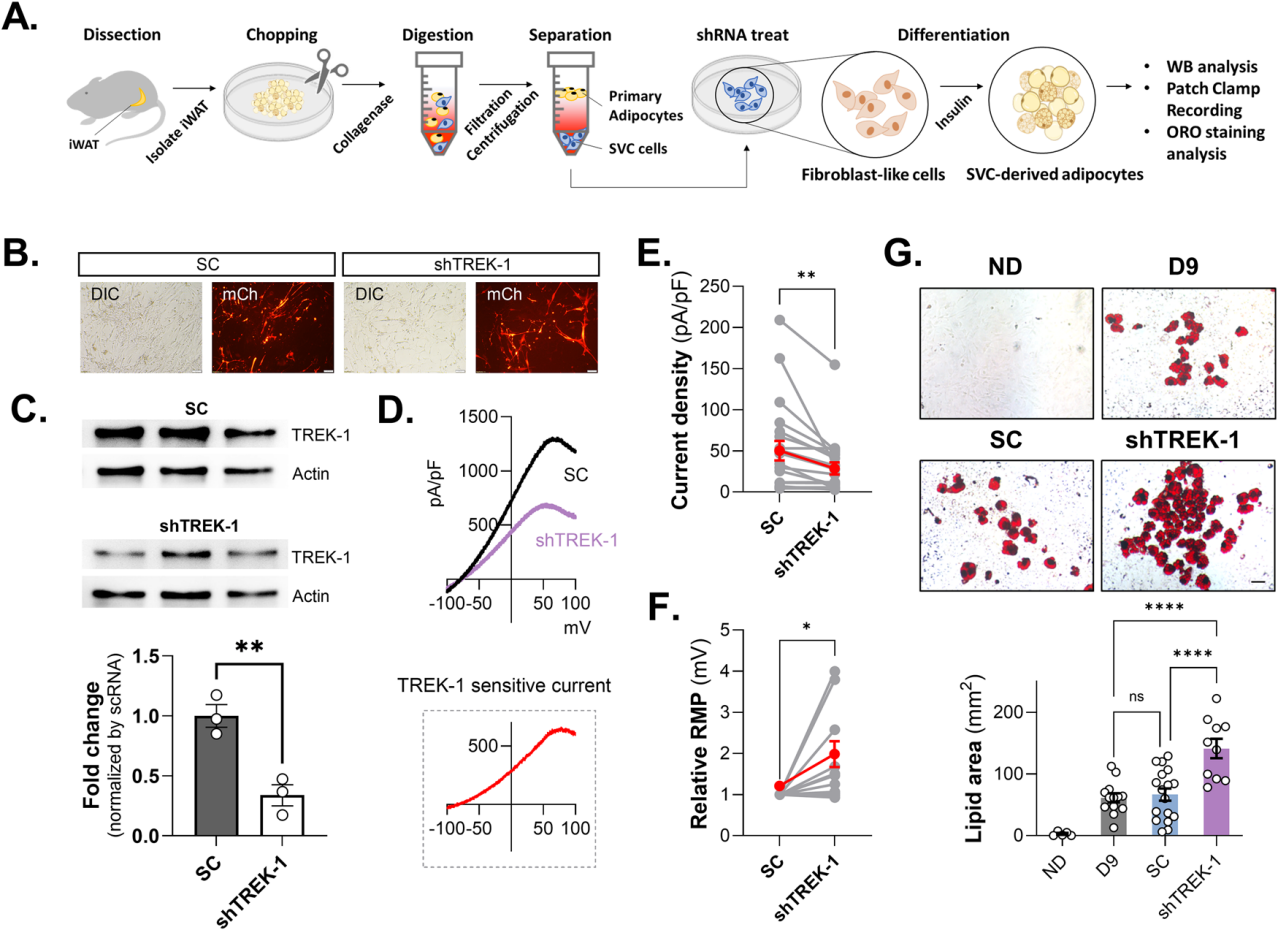
Increase of lipid accumulation in primary cultured preadipocytes by TREK-1 KD. **A** Schematic diagram of experimental procedure for primary adipocyte culture. **B** Example of transfection of SC and shTREK-1 into primary cultured preadipocytes, indicated by observable fluorescence. **C** WB analysis of TREK-1 KD by shTREK-1 transfection in primary cultured preadipocytes. **D** The comparison of current–voltage relationship (I–V) (from -100 mV to +100 mV) in primary cultured preadipocytes with shTREK-1. **E**, **F** Current density (pA/pF) and RMP changes in primary cultured preadipocytes with shTREK-1. **G** The results of the ORO staining and measurement of the lipid area in primary cultured adipocytes with shTREK-1 after the induction of differentiation.

We further investigated the role of TREK-1 in adipogenesis using primary adipocytes derived from inguinal white adipose tissue stromal vascular cells (iWAT SVCs) of C57BL/6 mice (Fig. 6A). Next, we confirmed that TREK-1 is expressed in iWAT SVCs using TREK-1 shRNA (Fig. 6B, C) and TREK-1 CRISPR/Cas9 transfection (Supplementary Fig. 8). To assess the functionality of TREK-1 in primary iWAT SVCs, we recorded voltage-induced ramp currents and a significant decrease in the K^+^ current was confirmed by the application of TREK1 shRNA (Fig. 6D, E). And also, we confirmed that TREK-1 KD induced depolarization of RMP in iWAT SVCs (Fig. 6F). Our results indicated the presence of functional TREK-1 channels in primary adipocytes. We transfected primary iWAT SVCs with TREK-1 shRNA and induced adipocyte differentiation to investigate the effect of TREK-1 on adipogenic differentiation. After nine days, we performed ORO staining to analyze lipid accumulation and observed a significant increase in lipid droplets in the shTREK-1 transfected group compared to the SC group (Fig. 6G). Our results suggest that TREK-1 plays a crucial role in primary iWAT SVCs and 3T3-L1 cells. The consistency between the ex vivo and in vitro results further strengthens the evidence supporting the role of TREK-1 in adipogenesis.

### HFD-fed TREK-1 KO mice increased adipogenic properties

**Fig. 7:**
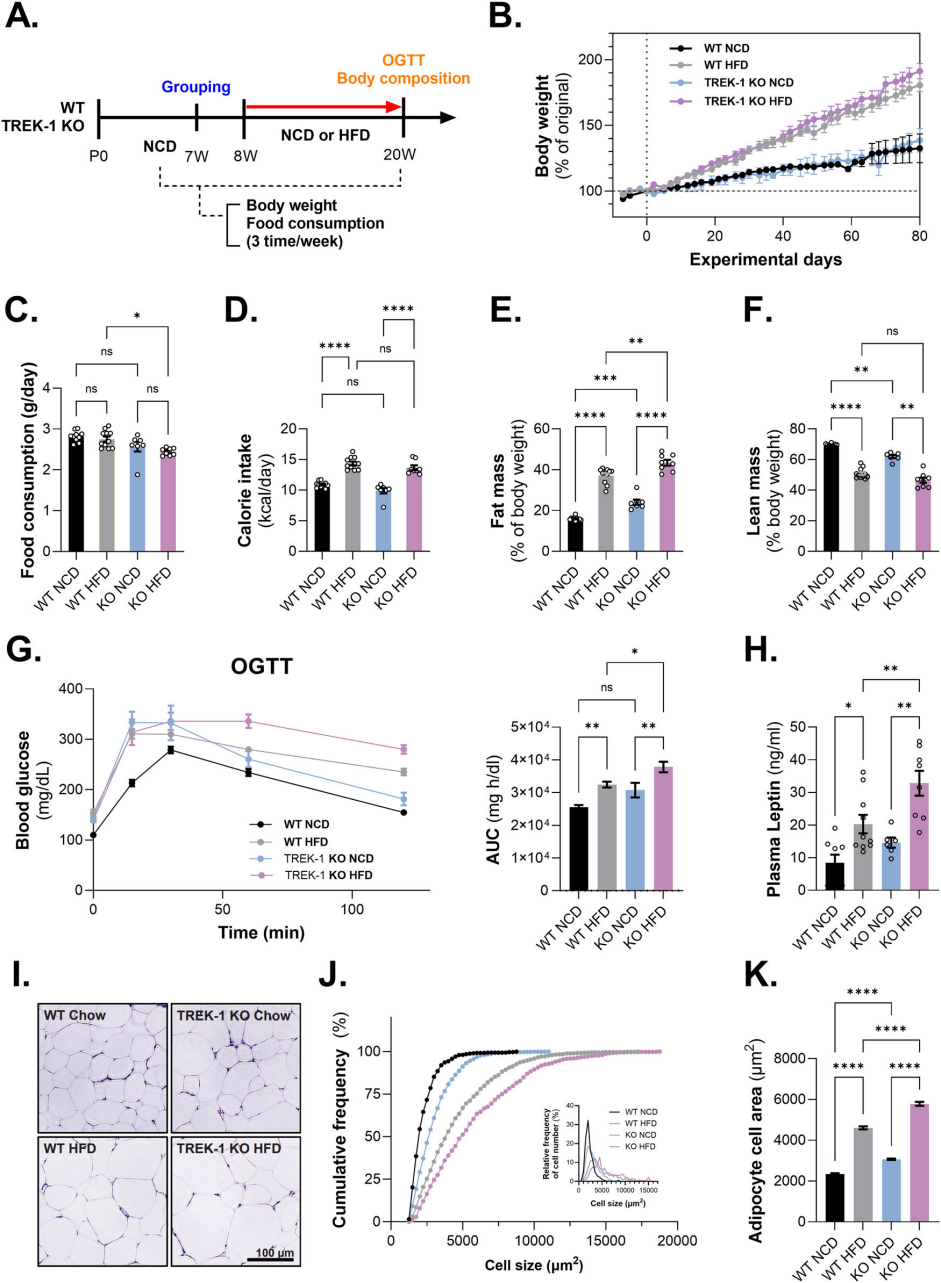
Metabolic alteration in WT or TREK-1 KO mouse with HFD consumption. **A** Schematic experimental design for animal feeding. **B** The body weight changes. **C** Food intake per day, **D** calorie intake per day, **E** % Fat mass, **F** % Lean mass, **G** Blood glucose level during OGTT and its AUC. **H** Plasma leptin level (ng/mL). **I** Histology of epididymal fat. **J** The cumulative frequency of adipocyte cell number. **K** The adipocyte cell area (%) depends upon the size of the adipocyte. One-way ANOVA to obtain *P*-values ****P < 0.0001. Data are mean ± s.e.m.

Next, to evaluate the systemic effects of TREK-1 on the development of obesity, we subjected wild-type (WT) and TREK-1 KO mice to either a normal chow diet (NCD) or a high-fat diet (HFD) from 8 to 20 weeks of age (Fig. 7A). Body weight gradually increased in the HFD groups of both WT and KO mice; however, there was no significant difference in the NCD group (Fig. 7B). Food intake decreased in TREK-1 KO mice fed an HFD compared to WT mice fed an HFD (Fig. 7C), but calorie intake did not show significant differences (Fig. 7D). Fat mass was significantly increased in the HFD groups of both mice. Interestingly, TREK-1 KO mice showed a significant increase in fat mass and a decrease in lean body mass, even in the NCD group (Fig. 7E, F). We also observed decreased glucose disposal in the oral glucose tolerance test (OGTT) in TREK-1 KO mice compared to WT mice on NCD and HFD (Fig. 7G). The plasma leptin level was significantly increased in KO mice fed HFD than in WT mice fed HFD (Fig. 7H). Additionally, elevated fasting glucose levels were observed in the TREK-1 KO without HFD compared to the WT control group (Supplementary Fig. 9). Furthermore, we confirmed that the level of acetyl-CoA carboxylase (ACC), enzyme regulating fatty acid synthesis and oxidation, were significantly decreased in TREK-1 KO mice when compared to WT mice. Additionally, we observed a significant reduction in adipoq (adiponectin) expression levels following HFD consumption in TREK-1 KO mice (Supplementary Fig. 10). In the HFD group of TREK-1 KO mice, we observed a significant increase in adipocyte size compared to that in WT mice. Adipocyte size and area were increased in TREK-1 KO mice fed an HFD (Fig. 7I–K). These results suggest that TREK-1 deficiency may play a role in the development of obesity and related metabolic disorders.

## Discussion

In this study, we observed that potassium currents and TREK-1 expression significantly decreased during the differentiation of preadipocytes into matured adipocytes. Blocking TREK-1 led to increased lipid accumulation and reduced adipogenic markers via PKCα modulation, suggesting TREK-1’s involvement in adipocyte maturation through intracellular calcium regulation. TREK-1 activation, in contrast, was found to inhibit lipid accumulation throughout adipogenesis. Notably, TREK-1-deficient mice fed a high-fat diet (HFD) exhibited greater fat mass and tended to amplified insulin resistance compared to wild-type mice. These findings suggest that TREK-1 functions as a key regulator of adipogenesis and may play a protective role against HFD-induced obesity and insulin resistance.

Adipogenesis is a complex process involving the differentiation of preadipocytes into mature adipocytes and is regulated by various signaling pathways, including the critical insulin signaling pathway [4]. In this study, we investigated the role of K2P ion channels, which are known to be important for the resting membrane potential in adipocyte differentiation, using 3T3-L1 preadipocytes. While previous studies have reported that TREK-2 may be involved in the early stages (3–6 h) of adipocyte differentiation [23] and that TASK-1 is important for the β-adrenergic response in brown adipocytes [32], no study has verified the expression of all K2P family members. We observed a decrease in potassium currents during adipocyte differentiation, and while the expression of all K2P ion channels was confirmed, only TREK-1 was selectively decreased. These findings align with recent analyses of K2P expression changes in transcriptome databases from human adipose tissue (human adipose tissue stem cells induced to differentiate into adipocytes for 9 days) (Supplementary Fig. 2) [17].

Among the many important molecules identified that are involved in adipogenesis, we found that AMPK phosphorylation was reduced during adipocyte differentiation, but the effect of TREK-1 inhibition was not significant once differentiation had progressed. AMPK is an important enzyme involved in the regulation of cellular energy balance. It is activated in response to metabolic stress and regulates intracellular energy homeostasis by modulating the ATP/AMP ratio. This is primarily achieved by inhibiting ATP-consuming processes while simultaneously promoting ATP production. In the context of adipogenesis, AMPK typically plays an initial role in inhibiting the differentiation of preadipocytes into adipocytes, indicating that AMPK functions as a negative regulator of lipid accumulation. It has been demonstrated that AMPK inhibits the activation of PPARγ, a key transcription factor essential for initiating adipogenesis [1, 33–35]. This is likely because AMPK plays a role early in differentiation, and treatment of adipocytes with Spadin before differentiation significantly reduced AMPK phosphorylation (Fig. 2G).

In reports, extracellular signal-regulated kinases (ERKs) are identified as playing a dual role in the process of adipogenesis, regulating key stages in the proliferation and differentiation of adipocytes. In the initial stages of adipogenesis, the activation of ERK stimulates the proliferation of adipocytes and elevates the transcription factors C/EBPβ and PPARγ, which subsequently facilitate cellular differentiation. However, in the subsequent stages of differentiation, the activity of ERK must be downregulated in order for the cells to fully mature. Prolonged ERK activation can maintain the cells in a proliferative state and inhibit differentiation [21, 36, 37]. However, our observations did not identify any clear trends during the differentiation process except D5 (Fig. 3E), leading us to hypothesize that the activation of cells by TREK-1 and the activation pathway of ERK may be distinct processes, which warrant further investigation in future studies.

During the process of adipogenesis, FABP4 also plays a pivotal role, particularly during the mid-to-late stages of adipocyte formation. In the initial stages, transcription factors such as PPARγ and C/EBPα become activated, establishing a preadipocyte differentiation phase with minimal to no expression of FABP4. However, at the mid-stage, there is a marked increase in FABP4 expression as a result of the influence of PPARγ. This facilitates intracellular fatty acid metabolism through fatty acid binding. By the late stage of differentiation, as the adipocytes reach maturity, FABP4 continues to be expressed at high levels, supporting the transport of fatty acids and maintaining metabolic balance within the cells. We detected upregulation of FABP4 during adipocyte differentiation (Fig. 3H). However, in contrast to our initial hypothesis, the observed increase did not continue upon treatment with Spadin. It can be reasonably deduced that this was the result of differentiation already being at an advanced stage, which meant that no further effect was observed in TREK-1 inhibition by Spadin treatment. These results therefore suggest that the reduction of TREK-1 expressed in adipose progenitor cells could be recognized as a marker of early adipogenesis, and that regulation of TREK-1 activity has potentially important implications in the development of obesity. Spadin appears to modulate the expression of the LAP isoform of CEBPβ through mechanisms potentially involving translational regulation or enhanced protein stability [38]. In this study, we confirmed that the expression of C/EBPβ (LAP) has been significantly increased in adipocyte differentiation by TREK-1 inhibition with Spadin (Supplementary Fig. 5). This robust our hypothesis that suppression of TREK-1 modulation promotes adipocyte differentiation.

The relationship between PKC and TREK-1 has been studied in neurons, where it has been reported that PKC regulates TREK-1 activity through PKC-dependent phosphorylation, which regulates cell excitability and calcium signaling in neurons [30, 39, 40]. TREK-1 plays a role in maintaining cellular hyperpolarization and resting membrane potential, thereby regulating neuronal excitability, neuroprotection, and pain modulation. Interestingly, the present study revealed that preadipocytes expressed TREK-1 and demonstrated that TREK-1 inhibition results in the depolarization of preadipocytes, thereby increasing the influx of intracellular calcium, which is a critical factor in cell differentiation and proliferation. In this context, Spadin, a TREK-1 inhibitor, was demonstrated to facilitate calcium influx in preadipocytes, an effect that was prevented by the L-type calcium channel blocker nifedipine. The increase in intracellular calcium during TREK-1 inhibition may be associated with elevated PKC activity, as PKC phosphorylation of TREK-1 is observed during adipocyte differentiation (Figs. 3 and 4). This suggests that TREK-1 inhibition may accelerate PKC-dependent pathways, influencing both calcium dynamics and cellular differentiation processes (Fig. 4A–C). Consequently, PKC-mediated inhibition of TREK-1 has substantial implications for adipogenesis in terms of cellular excitability, differentiation, and calcium-dependent signaling. These findings collectively indicate the existence of a positive feedback loop, whereby TREK-1 inhibition-induced depolarization results in increased intracellular calcium levels. Subsequently, elevated calcium concentrations activate PKC, which further inhibits TREK-1. This amplifies the initial depolarizing stimulus and contributes to sustained increases in intracellular calcium. The elevation of intracellular calcium levels in response to the increase of RMP, coupled with its reversal by PKC inhibition by GO6983, provides substantial support for this model (see Supplementary Fig. 11). Additionally, these results show that this process regulates adipogenesis by activating the expression of C/EBPα and PPARγ, which are critical transcription factors in adipogenesis [25, 41–43].

The current study has revealed a number of indicators of potential insulin resistance in TREK-1 knockout mice, including an observed decrease in ACC, and adiponectin (Supplementary Fig. 8), rise in OGTT and leptin levels, and enlargement of adipocyte size in TREK-1 knockout mice (Fig. 7G–K). ACC decline may imply that absence of TREK-1 did not induce adipocyte hyperplasia but could hypertrophy promoting lipid accumulation, while adiponectin reduction is closely linked with disrupted metabolic balance, leading to the development of insulin resistance (Supplementary Fig. 10). Increased OGTT results suggest impaired glucose metabolism, indicating possible insulin dysfunction. Additionally, higher leptin levels and increased adipocyte size could promote inflammatory responses in adipose tissue, further exacerbating insulin resistance. These findings collectively suggest that TREK-1 deficiency may contribute to insulin resistance. However, limitations include the lack of direct confirmation of insulin resistance and the possibility that each indicator may not uniformly impact all metabolic pathways. Furthermore, the use of a global KO model complicates the attribution of observed metabolic alterations specifically to adipose tissue. To address this, employing an adipose tissue-specific Cre model would allow a more precise analysis of TREK-1’s localized role, enabling a targeted study of its direct effects within adipose tissue.

TREK-1 is also a well-known mechano- and temperature-activated background potassium channel [44, 45]. It opens steadily and reversibly under heat or cold conditions [44]. A 10 °C increase enhances the TREK-1 current by approximately sevenfold [44, 46]. It has been reported that mechano-activated TRAKK and TREK-1 channels control both warm and cold sensations in polymodal pain perception [47, 48]. Unlike white fat, brown adipose tissue (BAT) can dissipate significant amounts of chemical energy through non-shivering thermogenesis [49, 50]. This process is mediated by major heat-generating factors, and BAT can be activated by specific stimuli such as cold exposure, adrenergic compounds, or genetic modifications [51]. It remains to be seen whether these properties of TREK-1 affect BAT. However, the mechano- and temperature-activated nature of TREK-1 suggests that TREK-1 may be involved in BAT thermogenesis, and future studies will need to address this.

The findings of this study highlight the specific impact of TREK-1 modulation on adipogenesis, particularly the contrasting effects of TREK-1 inhibition by Spadin and activation by ML402. Spadin, a selective TREK-1 antagonist, facilitated adipocyte differentiation by promoting lipid droplet accumulation and suppressing adipogenic markers, likely through the upregulation of PKCα and subsequent intracellular calcium influx. In contrast, ML402, a potent TREK-1 agonist, inhibited lipid accumulation and adipogenic progression at both early and late stages of adipocyte maturation. This dual regulatory approach—using TREK-1 inhibition to enhance and TREK-1 activation to suppress adipogenesis—provides a mechanistic basis for TREK-1’s role as a pivotal upstream modulator in adipocyte biology. The therapeutic application of ML402 highlights its potential to counteract accelerated adipogenesis, particularly under high-fat diet conditions, by stabilizing intracellular potassium flux and calcium homeostasis. Additionally, these results underscore TREK-1 activation as a promising intervention to modulate adipocyte expansion, reduce lipid accumulation, and attenuate metabolic disturbances associated with obesity and insulin resistance. Therefore, TREK-1 modulators, particularly activators like ML402, represent a targeted approach for therapeutic strategies aimed at mitigating adipogenesis-related metabolic pathologies.

## Supplementary information

Original WB images

Supplementary File

## Data Availability

The datasets used and/or analyzed during the current study are available from the corresponding author upon reasonable request.
