# Supplementary material for: Novel function of TREK-1 in regulating adipocyte differentiation and lipid accumulation
Source: Cell Death Dis. 2025 Mar 8;16(1):164. doi: 10.1038/s41419-025-07478-3 (PMC11890776; doi:10.1038/s41419-025-07478-3)

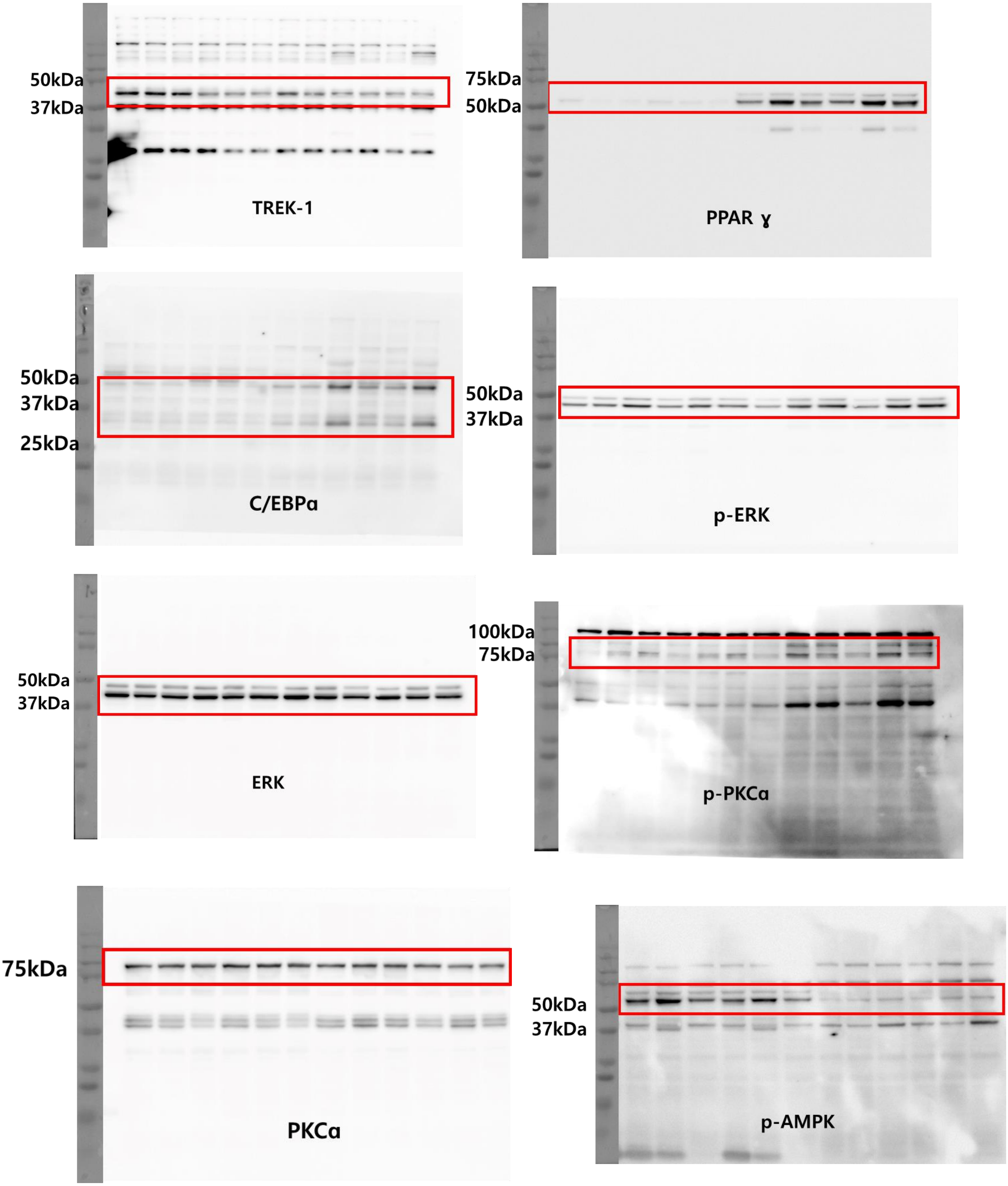
50kDa
37kDa
TREK-1
75kDa
50kDa
PPAR γ
50kDa
37kDa
25kDa
C/EBPα
50kDa
37kDa
p-ERK
50kDa
37kDa
ERK
100kDa
75kDa
p-PKCα
75kDa
PKCα
50kDa
37kDa
p-AMPK

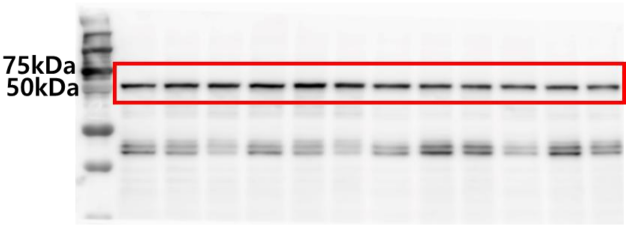

AMPK

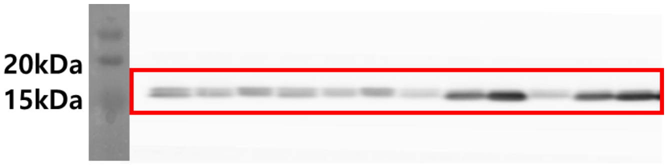

FABP4

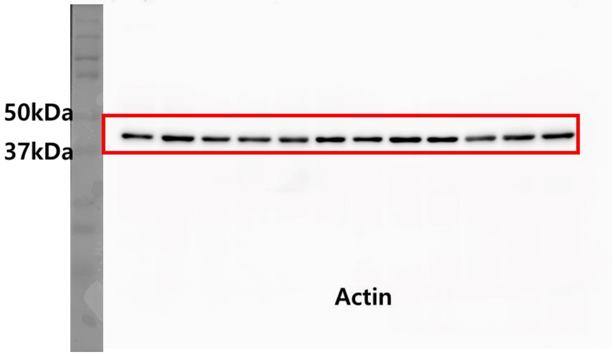

3T3L1_Spadin_day3,5,7 differentiation

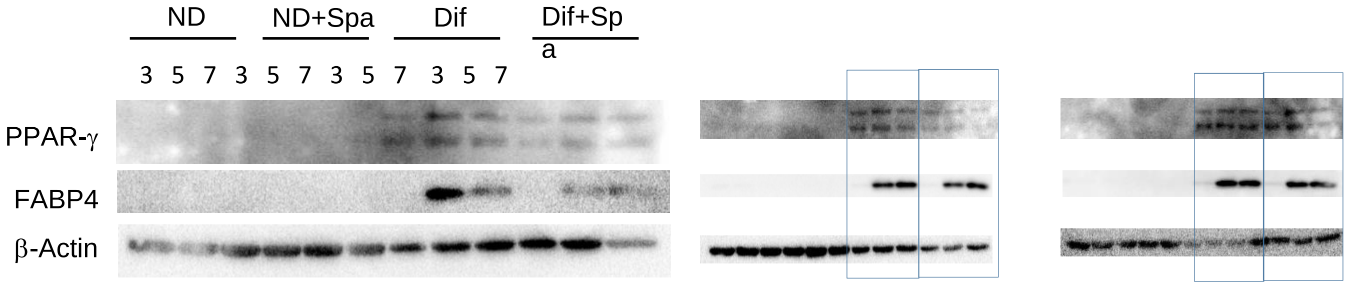

Blot1 Blot2 Blot3

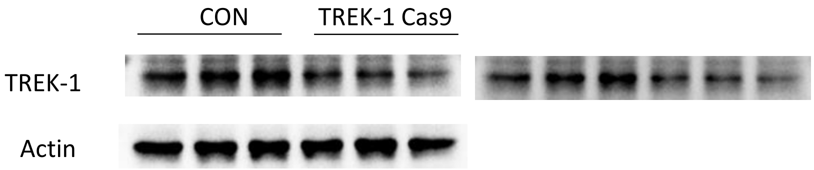

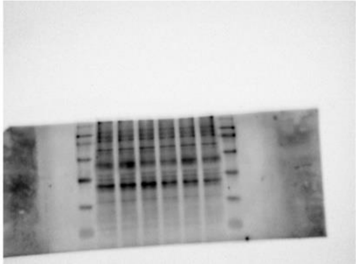

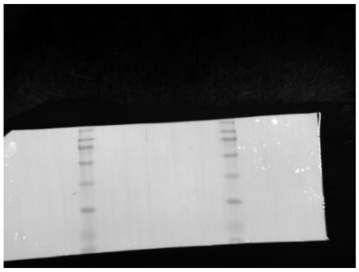

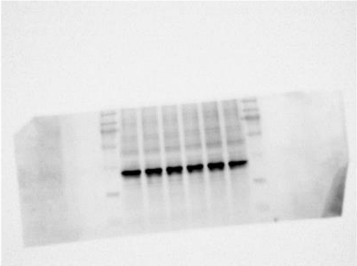

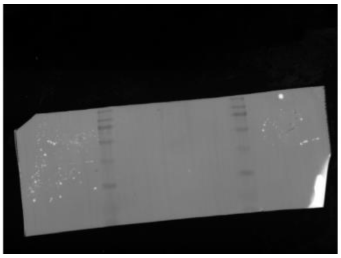

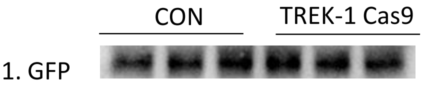

GFP (C2) : santa cruz biotechnology # sc-390937

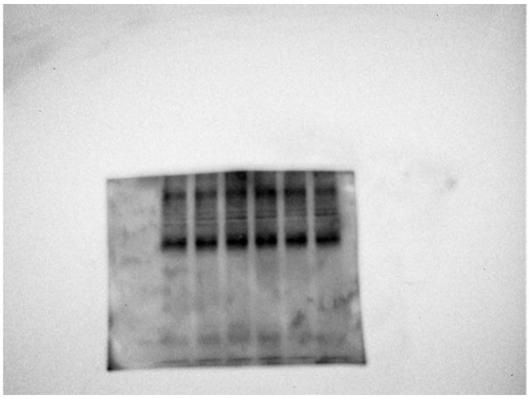

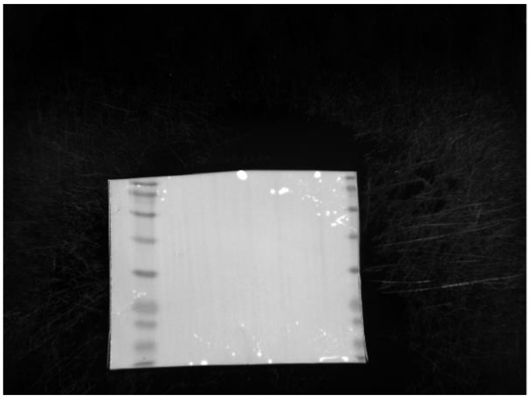

Spadin effect on C/EBPβ expression

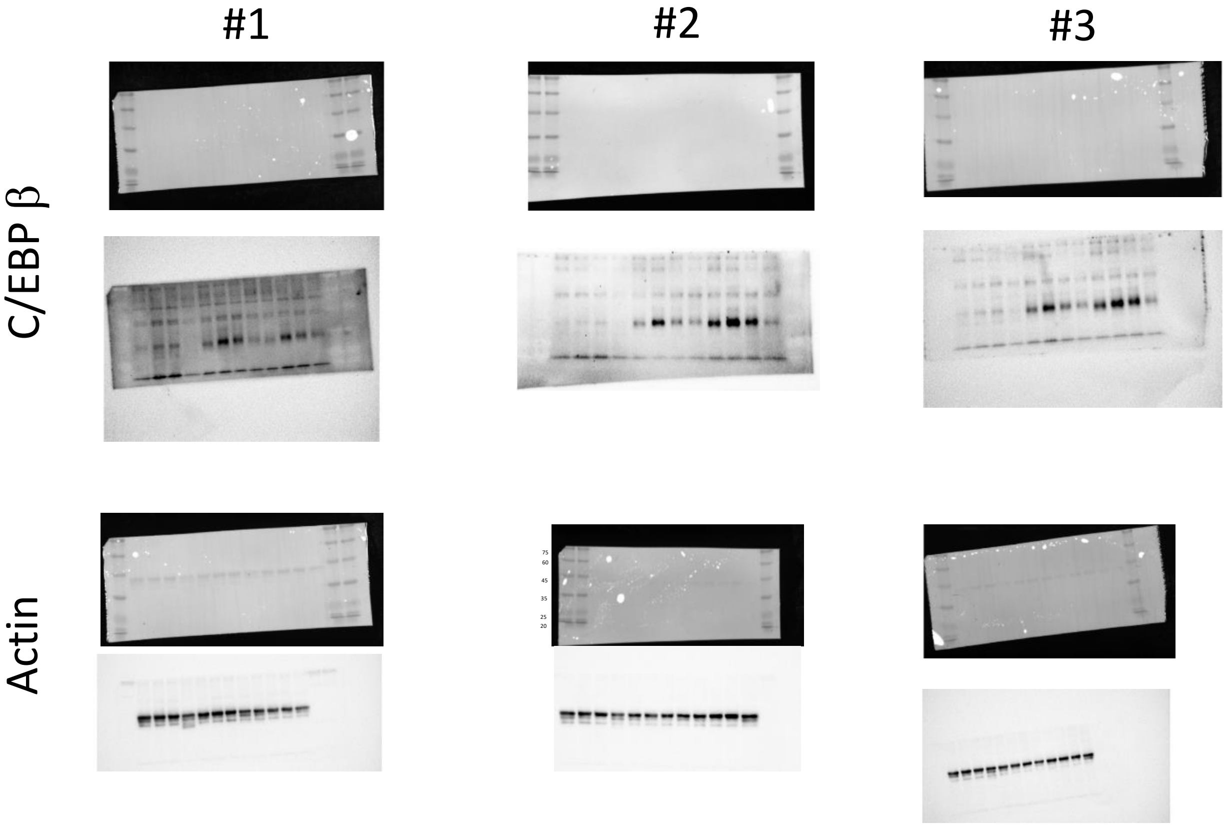

Supplement: Supplementary file 1 — Original WB images [file 41419_2025_7478_MOESM1_ESM.pdf]
