# Supplementary material for: Novel function of TREK-1 in regulating adipocyte differentiation and lipid accumulation
Source: Cell Death Dis. 2025 Mar 8;16(1):164. doi: 10.1038/s41419-025-07478-3 (PMC11890776; doi:10.1038/s41419-025-07478-3)
Supplement: Supplementary file 2 — Supplementary File [file 41419_2025_7478_MOESM2_ESM.docx]

**SUPPLEMENTARY INFORMATION**

**Novel Function of TREK-1 in Regulating Adipocyte Differentiation and Lipid Accumulation**

Running title: TREK-1-Mediated Calcium Signalling Controls Adipogenic Fate

Ajung Kim^1,2*^, Seoyeong Jung^1,3*^, Yongeun Kim^1*^, Jonghoon Jung^1^, Soomin Lee^2,3^, Hojin Lee^1,3^, Min Jung Kim^1^, Jae-Yong Park^3^, Eun Mi Hwang^2†^ & Jaekwang Lee^1†^

^1^ Food Functionality Research Division, Korea Food Research Institute, Wanju, 55365, Korea

^2^ Brain Science Institute, Korea Institute of Science and Technology, Seoul, 02792, Korea

^3^ School of Biosystems and Biomedical Sciences, College of Health Sciences, Korea University, Seoul 02841, Korea

* These authors contributed equally to this work

SUPPLEMENTARY FIGURES

***Count of Lipid droplet by ImageJ***

To quantify ORO-stained lipid droplets in our samples, we used ImageJ software. First, images were converted to 16-bit grayscale under the Image menu. Using Image – Adjust – Threshold, we set the threshold to a dark background and adjusted the brightness slider to highlight positive staining, optimizing separation of lipid droplets. After applying the threshold, we quantified droplets by selecting Analyze – Analyze Particles, setting size limits from 20 or 30 to infinity, and displaying results with outlines. This produced raw data on droplet count and area, which we verified for accuracy before exporting. Finally, we transferred the “Area” data from the Results window into Excel, renaming each column by image date for organized analysis.

***ShRNA transfection***

The target region of shRNA as follows: mouse TREK-1: 5′-GCGTGGAGATCTACGACAAGT-3’

Ref : Mi Hwang, E., Kim, E., Yarishkin, O. et al. A disulphide-linked heterodimer of TWIK-1 and TREK-1 mediates passive conductance in astrocytes. Nat Commun 5, 3227 (2014). <https://doi.org/10.1038/ncomms4227>

***Knock-out using the CRISPR/Cas9 system***

TREK-1 was knocked out in primary adipocytes using the CRISPR/Cas9 system. Control and TREK-1 CRISPR/Cas9 plasmids were purchased from Santa Cruz Biotechnology (Santa Cruz Biotechnology, cat #sc-421243 and #sc-421244, respectively). Briefly, plasmids were transfected into primary adipocytes via electroporation. The electroporated cells were seeded onto a culture plate and incubated for at least 24 hours. To evaluate the knock-out efficiency of TREK-1, the expression levels of TREK-1 (Santa Cruz Biotechnology, cat #sc-11556) and GFP (Santa Cruz Biotechnology, cat #sc-390394) were analyzed by Western blotting. Actin (Sigma-Aldrich, cat #A5441) was used as a loading control, and GFP expression was assessed to compare transfection efficiency.

**Supplementary Table 1.** **Real-time PCR primers and probes**

| Gene | Sequence |
| --- | --- |
| KCNK1  Forward Primer  Probe  Reverse Primer | 5’-CGGGAAATTGGAATTGGGAC-3’  5’-/56-FAM/CGAAGAAGA/ZEN/GCGCCGAGGTGA/3IABkFQ/-3’  5’-GATGCCGATGACAGAGTAGATG-3’ |
| KCNK2  Forward Primer  Probe  Reverse Primer | 5’- TGGCTACGGGTGATCTCTAAG-3’  5’-/56-FAM/CCGCCTGGT/ZEN/CGTTTCCTTGAACT/3IABkFQ/-3’  5’- GCTGGAACTTGTCGTAGATCTC-3’ |
| KCNK3  Forward Primer  Probe  Reverse Primer | 5’-CTCCTTCTACTTCGCCATCAC-3’  5’-/56-FAM/AGAACATGC/ZEN/AGAACACCTTGCCTCC/3IABkFQ/-3’  5’-GGCTCTGGAACATGACTAGTG-3’ |
| KCNK4  Forward Primer  Probe  Reverse Primer | 5’-TTTGGCGATTATGTACCCGG-3’  5’-/56-FAM/CAGAACTCT/ZEN/CCAGCCTACCAGCC/3IABkFQ/-3’  5’-AAGATCCAGAACCACACCAG-3’ |
| KCNK5  Forward Primer  Probe  Reverse Primer | 5’-TCATCACCATCTCCACCATTG-3’  5’-/56-FAM/CCCAGGTAG/ZEN/ATCCAAAGCTCCACAA/3IABkFQ/-3’ 5’-GGCTCTGGAACATGACTAGTG-3’ |
| KCNK6  Forward Primer  Probe  Reverse Primer | 5’-GGCAGTCAGAAGTAGCATGG-3’  5’-/56-FAM/AGTGCAAAG/ZEN/ACGATGGAGAAGGCTT/3IABkFQ/-3’  5’-AGCACGCTTGTCACCAC-3’ |
| KCNK9  Forward Primer  Probe  Reverse Primer | 5’-TTCCTTCTACTTCGCCATCAC-3’  5’-/56-FAM/CCCAGCACA/ZEN/GCGTAGAACATACAGA/3IABkFQ/-3’ 5’-TGGAACATAACCAGCGTCAG-3’ |
| KCNK10  Forward Primer  Probe  Reverse Primer | 5’-GTAGGCTTTGGTGATTTTGTGG-3’  5’-/56-FAM/CGCTGGTGT/ZEN/GGTTTTGGATCCTTG/3IABkFQ/-3’ 5’-AGTCTCCGATCATACTGAGGAC-3’ |
| KCNK12  Forward Primer  Probe  Reverse Primer | 5’-GAACAGTCCGTAGGCGATG-3’  5’-/56-FAM/TGTCATGCC/ZEN/GAAACCTATGGTCGAC/3IABkFQ/-3’ 5’-CTGGAGCCTTCTACTTCGTG-3’ |
| KCNK13  Forward Primer  Probe  Reverse Primer | 5’- GGTTGAAGAAGAGGATGGTACT-3’  5’ -/56-FAM/CCGTTGTGG/ZEN/CTGGTGTTGTCATC/3IABkFQ/-3’  5’-CTTCGTGGGTACAGTGGTTT-3’ |
| KCNK16  Forward Primer  Probe  Reverse Primer | 5’- GATTGGTGGAGTTGCCTTTG-3’  5’-/56-FAM/AGGTCATCC/ZEN/TGGAAGCCTGGG/3IABkFQ/-3’  5’-AGAACTACACCTGCCTAGACC-3 |
| KCNK18  Forward Primer  Probe  Reverse Primer | 5’-GGCTTGAGGTGTTGCAGAT-3’  5’-/56-FAM//ZEN//3IABkFQ/-3’  5’-ACCCTGAGTTGAAGAAGTTCC-3 |
| GAPDH  Forward Primer  Probe  Reverse Primer | 5’-GTGGAGTCATACTGGAACATGTAG-3’  5’-/56-FAM/TGCAAATGG/ZEN/CAGCCCTGGTG/3IABkFQ/-3’  5’-AATGGTGAAGGTCGGTGTG-3’ |

**Supplementary Figure 1.**

**
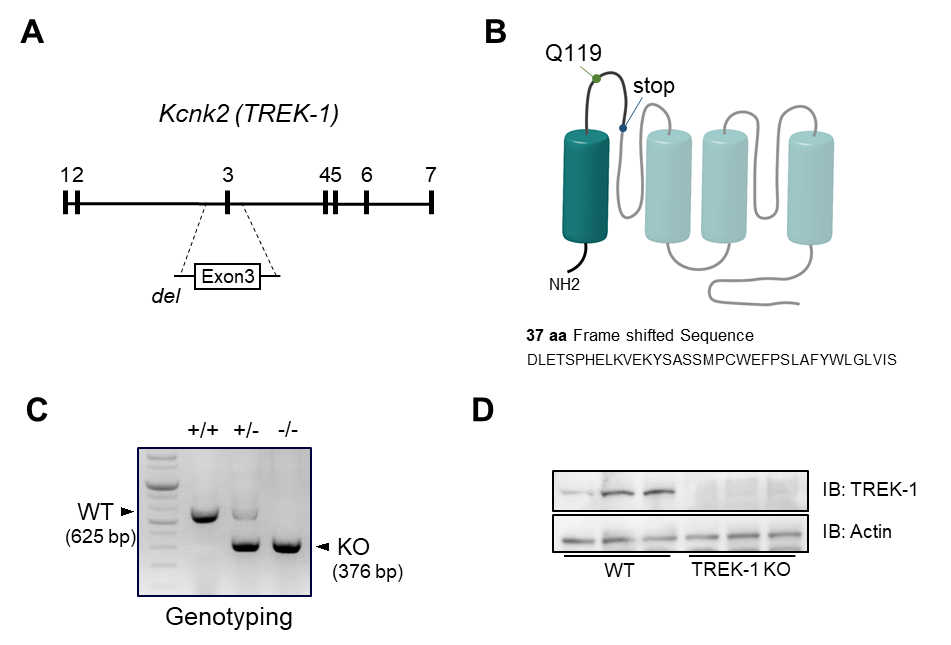
**

**Supplementary figure 1.** **Generation of TREK-1 knockout (KO) mice and validation in adipose tissue of TREK-1 KO mice.** (A) Schematic representation of the TREK-1 genomic structure: Exon1-7 are represented by the numbered, filled boxes. Exon 3 was removed for KO construction. (B) Schematic diagram of proteins predicted to be expressed by knockout of TREK-1. (C) Genotyping analysis from iWAT tissue of wild-type, heterozygous, or homozygous TREK-1 KO mice. (D) Western blot analysis to verify protein expression in WT and TREK-1 KO mice.

**Supplementary Figure 2.**


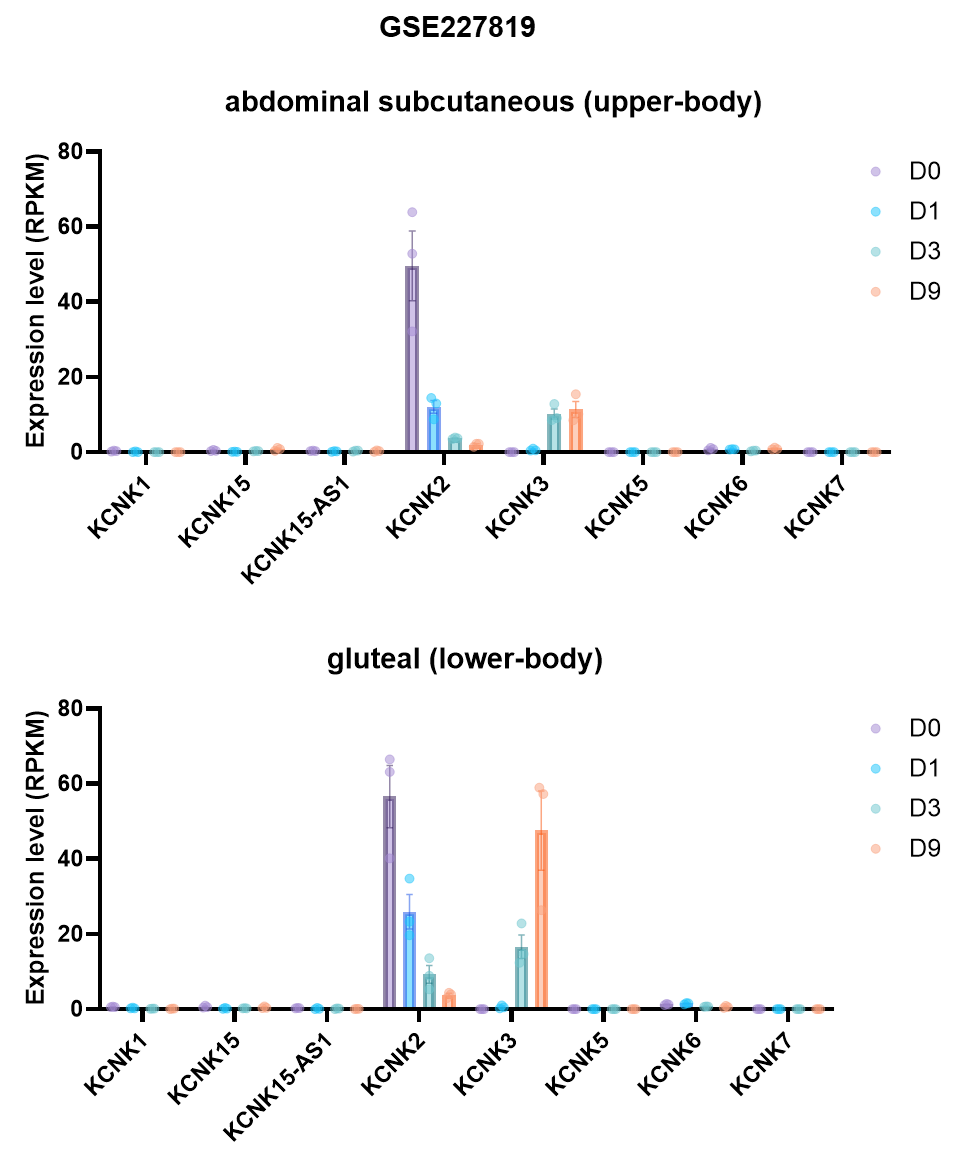


**Supplementary figure 2.** Changes in mRNA expression of K2P ion channels over time in adipocytes differentiated from human adipose stem cells. The mRNA expression of K2P ion channels was reanalyzed from the RNAseq dataset of GSE227819 and plotted as RPKM values.

**Supplementary Figure 3.**


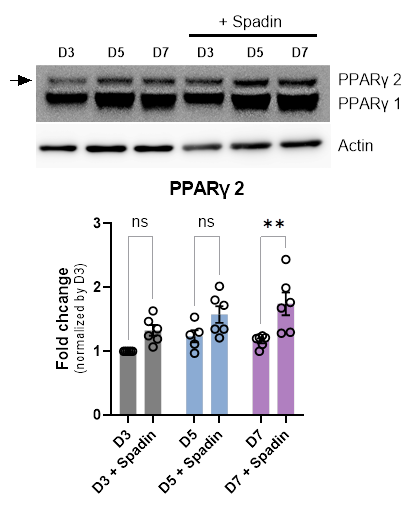


**Supplementary figure 3. Effect of TREK-1 inhibition on PPARγ2 isoform during adipocyte differentiation in 3T3L1 cell line.** Upper image indicates WB of PPARγ2 and γ1 at differentiation stages (D3, D5 and D7) with treatment of Spadin. The lower bar graph indicates the normalized fold change of PPARγ2. One-way ANOVA to obtain p-values **, p<0.01

**Supplementary Figure 4.**

**Supplementary figure 4. The effect of TREK-1 silencing on lipid accumulation and the expression of adipogenic markers in 3T3L1 cells.** (A) Example of transfection of scRNA and shTREK-1 in 3T3L1 cells after the induction of differentiation. (B-C) WB images and analysis of adipogenic markers (PPARγ, C/EBPα, and FABP4) with scRNA and shTREK-1 in 3T3L1 cells 5 days after differentiation (D5). (D-E) ORO staining images and quantification graph with scRNA and shTREK-1 in 3T3L1 cells 5 days after differentiation (D5). (F) Cell viability assay with lipofectamine

**Supplementary Figure 5.**


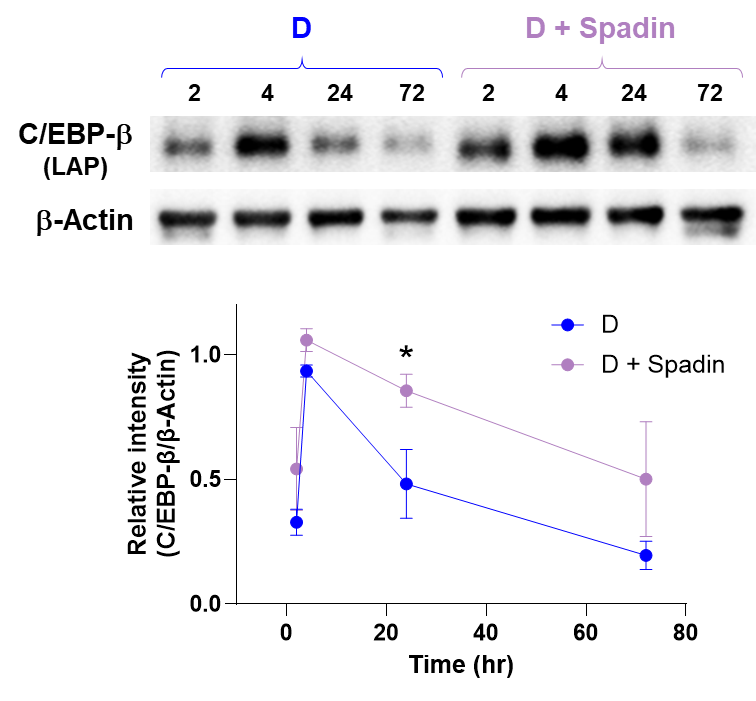


**Supplementary figure 10. Alteration of C/EBPβ expression by TREK-1/2 antagonist (Spadin) during adipogenesis differentiation in 3T3L1 cell line.** Upper image indicates WB of C/EBPβ at differentiation stages in time dependent manner (2, 4, 24, and 72 hours) with treatment of Spadin. The lower graph indicates the normalized relative intensity of C/EBPβ. One-way ANOVA to obtain p-values *, p<0.05

**Supplementary Figure 6.**

**Supplementary figure 5. PPARγ and** **C/EBPα expression by TREK-1 antagonist (Spadin) and calcium channel blocker (Nifedipine) during adipogenesis differentiation in 3T3L1 cell line.** mRNA expression of PPARγ and C/EBPα at differentiation stages at differentiation stages (3, 5 and 7 days) with treatment of Spadin(?) or Spadin with Nifedipine(?). The lower graph indicates the normalized fold change of PPARγ and C/EBPα by GAPDH. One-way ANOVA to obtain p-values ****P<0.0001.

**Supplementary Figure 7.**


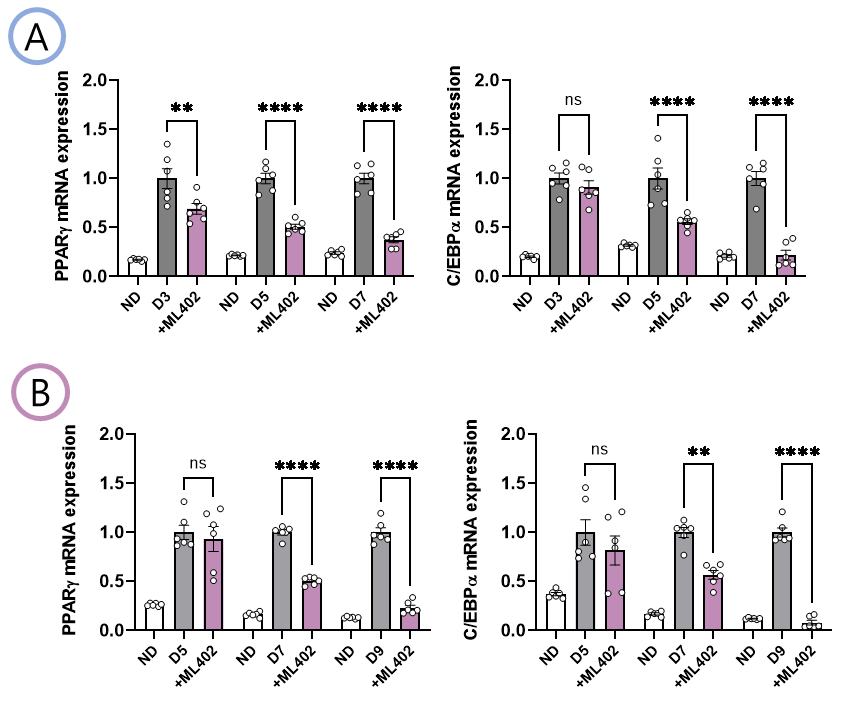


**Supplementary figure 6.** **PPARγ and** **C/EBPα expression by TREK-1 agonist (ML402) during adipogenesis differentiation in 3T3L1 cell line.** mRNA expression of PPARγ and C/EBPα at differentiation stages at differentiation stages (3, 5 and 7 days for ‘A’, 5, 7, and 9 days for ‘B’) with treatment of ML402. (A) ML402 (?) was treated from day 0 to day 7 during adipogenesis differentiation. (B) ML402 (?) was treated from day 3 to day 9 during adipogenesis differentiation. The lower graph indicates the normalized fold change of PPARγ and C/EBPα by GAPDH. One-way ANOVA to obtain p-values ** P < 0.01 and ****P<0.0001.

**Supplementary Figure 8.**


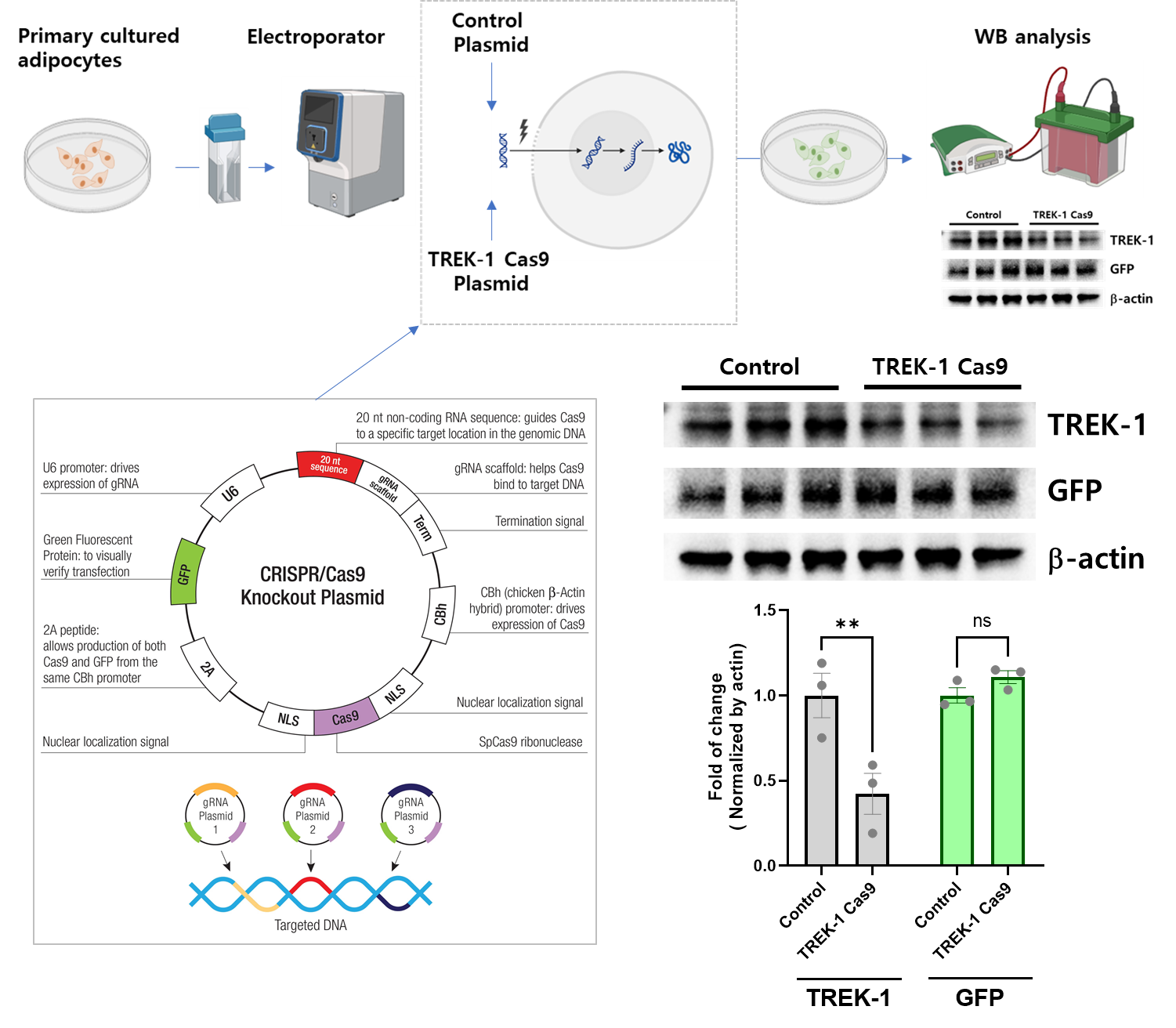


**Supplementary figure 7. Validation of TREK-1 Cas9 manipulation.** (Left) The map of CRISPR/Cas9 system. (Right) Upper image indicates validation of TREK-1 Cas9 in primary adipocyte. The lower bar graph indicates the normalized fold change of TREK-1. Student t-test to obtain p-values *, p<0.05

**Supplementary Figure 9.**


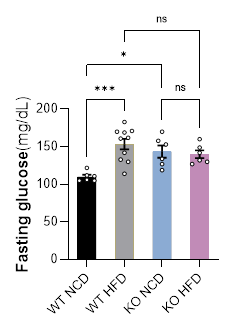


**Supplementary figure 8.** Analysis of fasting glucose level in WT and TREK-1 KO mice.

**Supplementary Figure 10.**


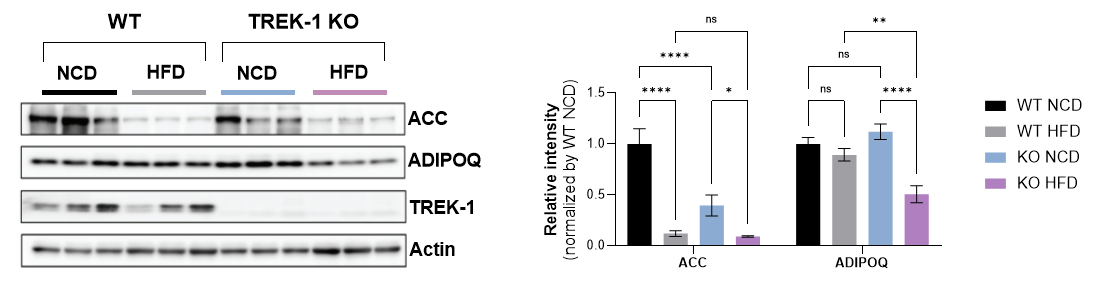


**Supplementary figure 9**. **Analysis of change of adipogenic marker in WT and TREK-1 KO mice with HFD.** WB images (left) and analysis graph (right) confirming the expression of ACC and Adipoq in adipose tissue (iWAT) of WT and TREK-1 KO mice fed NCD and HFD, respectively. Note: TREK-1 antibody was used to validate KO.

**Supplementary Figure 11.**


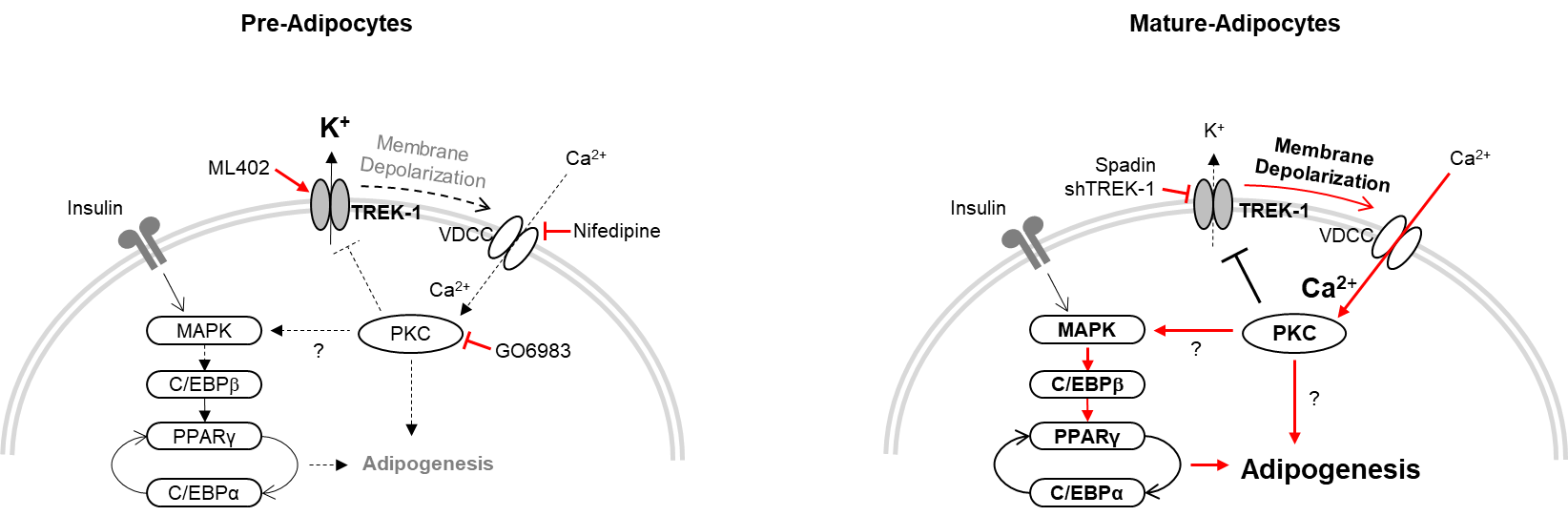


**Supplementary figure 11.** **Schematic diagram of the mechanism of TREK-1 associated with adipogenesis.** (Left) Prior to differentiation, adipocytes exhibit high level of TREK-1 expression on their cell membranes. This maintains a stable membrane potential, which in turn prevents the progression of adipogenesis. (Right) In mature-adipocytes, there is a decrease in TREK-1 expression as differentiation progresses, which results in a blockage of K^+^ movement, which causes an increase in membrane potential. This subsequently induces the activation of voltage-dependent calcium channels (VDCC), which in turn activates PKC. The activated PKC acts in a negative feedback loop to inhibit the activity of TREK-1, thereby accelerating the progression to adipogenesis.
